# Zeb2 Regulates Cell Fate at the Exit from Epiblast State in Mouse Embryonic Stem Cells

**DOI:** 10.1002/stem.2521

**Published:** 2016-11-08

**Authors:** Agata Stryjewska, Ruben Dries, Tim Pieters, Griet Verstappen, Andrea Conidi, Kathleen Coddens, Annick Francis, Lieve Umans, Wilfred F. J. van IJcken, Geert Berx, Leo A. van Grunsven, Frank G. Grosveld, Steven Goossens, Jody J. Haigh, Danny Huylebroeck

**Affiliations:** ^1^ Department of Development and RegenerationKU LeuvenLeuven3000Belgium; ^2^ Department of Cell BiologyErasmus University Medical CenterRotterdam3015 CNThe Netherlands; ^3^ Center for Biomics, Erasmus University Medical CenterRotterdam3015 CNThe Netherlands; ^4^ VIB Inflammation Research Center (IRC), Unit Vascular Cell Biology; ^5^ Department of Biomedical Molecular Biology; ^6^ VIB‐IRC, Unit Molecular and Cellular OncologyGhent UniversityGhent9052Belgium; ^7^ Center for Medical Genetics, Ghent University HospitalGhent9000Belgium; ^8^ Department of Cell BiologyLiver Cell Biology Lab, Vrije Universiteit BrusselJette1090Belgium; ^9^ ACBD ‐ Blood Cancers and Stem CellsGroup Mammalian Functional Genetics, Monash UniversityMelbourneVIC3004Australia

**Keywords:** Cell differentiation, DNA‐methylation, Embryonic stem cells, Pluripotent stem cells, Repressors, RNA‐sequencing, Transcription factors, Transcriptom

## Abstract

In human embryonic stem cells (ESCs) the transcription factor Zeb2 regulates neuroectoderm versus mesendoderm formation, but it is unclear how Zeb2 affects the global transcriptional regulatory network in these cell‐fate decisions. We generated *Zeb2* knockout (KO) mouse ESCs, subjected them as embryoid bodies (EBs) to neural and general differentiation and carried out temporal RNA‐sequencing (RNA‐seq) and reduced representation bisulfite sequencing (RRBS) analysis in neural differentiation. This shows that Zeb2 acts preferentially as a transcriptional repressor associated with developmental progression and that *Zeb2* KO ESCs can exit from their naïve state. However, most cells in these EBs stall in an early epiblast‐like state and are impaired in both neural and mesendodermal differentiation. Genes involved in pluripotency, epithelial‐to‐mesenchymal transition (EMT), and DNA‐(de)methylation, including *Tet1*, are deregulated in the absence of Zeb2. The observed elevated Tet1 levels in the mutant cells and the knowledge of previously mapped Tet1‐binding sites correlate with loss‐of‐methylation in neural‐stimulating conditions, however, after the cells initially acquired the correct DNA‐methyl marks. Interestingly, cells from such *Zeb2* KO EBs maintain the ability to re‐adapt to 2i + LIF conditions even after prolonged differentiation, while knockdown of Tet1 partially rescues their impaired differentiation. Hence, in addition to its role in EMT, Zeb2 is critical in ESCs for exit from the epiblast state, and links the pluripotency network and DNA‐methylation with irreversible commitment to differentiation. Stem Cells *2017;35:611–625*

**Significance Statement**

The transcription factor Zeb2 is critical for exit from the epiblast state in mouse ESCs and for neural and general differentiation. In addition to its role in EMT it links the pluripotency network and DNA‐methylation with irreversible commitment to differentiation.

## Introduction

Naïve mouse embryonic stem cells (mESCs), primed epiblast stem cells (EpiSCs), and embryonic germ cells are pluripotent cells that can be used as cell culture models to study pluripotent cell states and fate decisions that occur during embryogenesis 1, 2, 3, 4, 5, 6, transitions that require changes of the transcriptome and methylome. The ground state of self‐renewing mESCs can be achieved by simultaneous addition of chemical inhibitors (of MAPK and GSK3 signaling) and LIF (referred to as 2i + LIF) 7. When compared to a population of naïve embryonic stem cells (ESCs), ground‐state ESCs display higher and more homogeneous expression of key pluripotency genes, lower levels of differentiation markers and reduced DNA‐methylation 8, 9.

DNA‐methylation status has profound effects on embryonic gene expression. It is controlled by DNA (cytosine‐5)‐methyltransferases (Dnmt3a/3b/3l) that are highly active in ESCs and early embryos and establish new methylation patterns and by Dnmt1 that copies the patterns onto daughter cells 10, 11. Active demethylation is orchestrated by Ten‐eleven translocation methylcytosine dioxygenases (Tet) 12, 13. Tet1 levels are high in ESCs and decrease upon differentiation, correlating with exit from pluripotency, and Tet1 steers mesendoderm versus trophectoderm decisions in preimplantation embryos 14, 15. Tet1 is also important during somatic reprogramming for genome demethylation as well as activation/maintenance of Oct4 and Nanog 16, 17, 18.

Zeb2 (Sip1, Zfhx1b) downregulates *E‐cadherin* (*Cdh1*) and thereby steers epithelial‐to‐mesenchymal transition (EMT) 19, which is relevant to stem cell fate, but also tumorigenesis 20, 21. Mutations in *ZEB2* cause Mowat‐Wilson syndrome (MOWS; OMIM#235730), including defects in the central and peripheral nervous system (CNS, PNS) 22, 23, 24. Many in vivo studies confirm the critical roles of Zeb2 in embryogenesis and neurodevelopment in particular. *Zeb2* KO mice die shortly after E8.5 and have multiple defects, including in somitogenesis 25, the neural plate and neural crest cells 26. Cell‐type specific *Zeb2* KO mice develop defects in, for example, the CNS 27, 28, 29 and PNS 30, 31, 32. Such studies in embryonic brain revealed cell autonomous, but also non‐autonomous Zeb2 actions. In human (h) ESCs, Zeb2 regulates cell fate: upon Zeb2 knockdown (KD) they commit toward mesendoderm, while Zeb2 overproduction enhances neurogenesis 33. *ZEB2* is controlled by Nanog, Oct4, and Sox2 in hESCs, but key genes downstream of Zeb2 in ESCs, and during early neural development, remain to be determined, and *Zeb2* KO hESCs have not been reported. In order to enter lineage commitment, the pluripotency network in ESCs and EpiSCs needs to be distinguished 34, 35. The list of factors promoting exit from naïve or ground state is growing, yet more key players remain to be identified 36, 37, 38. Exit from pluripotency beyond the primed epiblast state requires efficient, irreversible silencing of the transcriptional pluripotency network (including *Oct4* and *Nanog* silencing, which persist in EpiSCs), acquisition and maintenance of DNA‐methyl marks, and initiation of differentiation.

Using *Zeb2* KO ESCs, we identified Zeb2 as a critical player for initiating and executing the differentiation programs. Upon withdrawal of 2i + LIF from *Zeb2* KO ESC populations, some cells only sometimes commit to differentiation, but instead the gross population usually stalls as pluripotent, epiblast‐like cells that maintain the ability to re‐adapt to 2i + LIF even after prolonged exposure to differentiation protocols. The defective silencing of the pluripotency program prevents these *Zeb2* KO cells from undergoing neural and general (including mesendodermal) differentiation. RNA‐seq revealed that Dnmt and Tet family mRNA levels are deregulated in *Zeb2* KO cells. Such cells correctly acquire methyl marks early during neural differentiation (ND), but do not maintain these and revert to a more naïve methylome state. Tet1 levels depend on the presence of Zeb2 and in *Zeb2* KO cells (displaying elevated Tet1) Tet1 KD rescues their ability to exit from their pluripotent state and re‐enter lineage commitment.

## Materials and Methods

### ESC Lines

All experiments on live mice used for deriving embryos for establishing the ESCs were performed in the Leuven lab according to institutional (KU Leuven P153/2012), national (lab license LA1210584, Belgian government) and international (2010/63/EU) guidelines and regulations. KU Leuven approved the experiments and confirmed that all experiments were done conform to the regulatory standards.

Two independent ESC derivations were performed. First, control lines were derived by interbreeding *Zeb2^flox/flox^* CD1 mice 39. Blastocysts were plated on mitomycin‐C inactivated mouse embryonic fibroblasts (mitC‐MEFs) in ESC derivation medium + LIF, and allowed to attach, and were re‐fed daily. After 5–6 days, the inner cell mass was separated from the trophectodermal layer, trypsinized and replated on mitC‐MEFs. They were further grown until subconfluency and expanded. From these ESCs, *Zeb2* KO lines were derived by nucleofection of linearized, blasticidin‐selectable (48 hours) pcDNA6‐His‐eGFP:Cre vector to low‐passage ESCs using Amaxa A‐23 (Lonza, Braine‐l'Alleud, BE, www.lonza.com). Five control ESC lines and two KO lines, confirmed as such by genotyping (details available on request), were established. Second, *Zeb2^+/‐^* mice were crossed with R26‐iPSC mice that contain a RMCE cassette in the ROSA26 (R26) locus 40. The second R26 allele contained the LacZ reporter 41. New control and RMCE‐compatible *Zeb2* KO ESC lines (three clones; mixed 129/Bl6 background) were derived using a protocol 42 in which pluripotin was replaced with 1 µM PD0325901 and 3 µM CHIR99021. To obtain R26_Zeb2 lines, RMCE technology 43 was used to insert N‐terminally Flag epitope (Flag) tagged, wild‐type Zeb2 cDNA into *R26* of *Zeb2* KO ESCs.

### ESC Cultures and Sorting

*ESC maintenance*: ESCs were maintained feeder‐cell free in 2i + LIF medium. N2B27 was prepared as described 44. For 2i + LIF medium, 1 μM PD0325901 (Axon, 1408, Axon Medchem, Groningen, NL, www.axonmedchem.com), 3 μM CHIR99021 (Axon, 1386) 7, and 1,000 U LIF/ml (Millipore, ESG1107, Merck Millipore, Zwijndrecht, BE, www.merckmillipore.com) were added. *Directed neural differentiation*: On d0, 3 × 10^6^ ESCs were plated in a 10‐cm bacterial petri dish in embryoid body (EB) medium (KO DMEM (Invitrogen, 10829018, Thermo Fisher Scientific, Merelbeke, BE, www.thermofisher.com), 15% fetal bovine serum (FBS, Life Technologies, 10270106, Thermo Scientific, Aalst, BE, www.fishersci.be/be), 0.1 mM nonessential amino acids (NEAA), 1 mM sodium pyruvate, 0.1 mM 2‐mercaptoethanol, 50 U/ml of penicilline/streptomycine, P/S). On d2 the EB medium was refreshed; on d4 it was changed to N2B27 + retinoic acid (Sigma‐Aldrich, R2625, Overijse, BE, www.sigmaaldrich.com; 500 nM) and refreshed on d6. Between d8 and d15 EBs were cultured in N2B27, which was refreshed every other day. *General EB differentiation*: On d0, 3 × 10^6^ ESCs were plated in a 10‐cm dish in EB medium (KO DMEM Invitrogen, 10829018), 10% FBS, 0.1 mM NEAA, 1 mM sodium pyruvate, 0.1 mM 2‐mercaptoethanol, 50 U/ml of P/S and changed every other day till d15. EBs on d15 were dissociated using Liberase (Roche, 05401020001, Roche Biochem Reagents, Overijse, BE, www.sigmaaldrich.com). Living cells were stained with propidium iodide (Sigma‐Aldrich, P4864) shortly before sorting. *ESC‐to‐EpiLC conversion*: ESCs were differentiated according to Hayashi et al. 45. Briefly 10^5^ ESCs were plated per well of a 12‐well plate coated with fibronectin (16.7 μg/ml, Millipore, FC010) in N2B27 containing Activin A (20 ng/ml, Peprotech, 120‐14E, Peprotech via Bio‐Connect, Huissen, NL, www.peprotech.com), bFGF (12 ng/ml, Peprotech, 100‐18C), and KSR (1%, Gibco, 10828010, Gibco, Merelbeke, BE, www.thermofisher.com). Medium was changed after 24 hours. The EpiLCs were collected after 48 hours.

### shRNA‐Mediated Knockdown

Control shRNA was used by combining MISSION Target shRNA in control vector SHC002 (Sigma‐Aldrich). The Tet1 shRNA (shTet: 5′‐tcatctacttctcacctagtg‐3′) was cloned into pLKO1. Control and Tet1 lentiviruses were produced by standard methods (see www.addgene.org/tools/protocols/pLKO).

### Chromatin Immunoprecipitation

10^7^ ESCs (from the R26_Zeb2 line) were used per experiment. Cells were cross‐linked for 10 minutes with ice‐cold 1% formaldehyde, sonicated using a Branson Digital Sonifier (10 pulses, 30 seconds ON; 60 seconds OFF, amplitude 10). 10 µg of anti‐Flag (Sigma‐Aldrich, F3165) and 10 µg of control mouse IgG (Santa Cruz, sc‐2025, Santa Cruz Biotechnology via Bio‐Connect, Huissen, NL, www.scbt.com) were used. Chromatin isolation and chromatin immunoprecipitation (ChIP) were done as described 46. Phenol‐chloroform purified DNA was used as template for qPCR to amplify the proximal promoters of *Nanog* and *Cdh1*. For primers, see Supporting Information Table SII.

### Immunohistochemistry and Indirect Immunofluorescence

**Figure 1:**
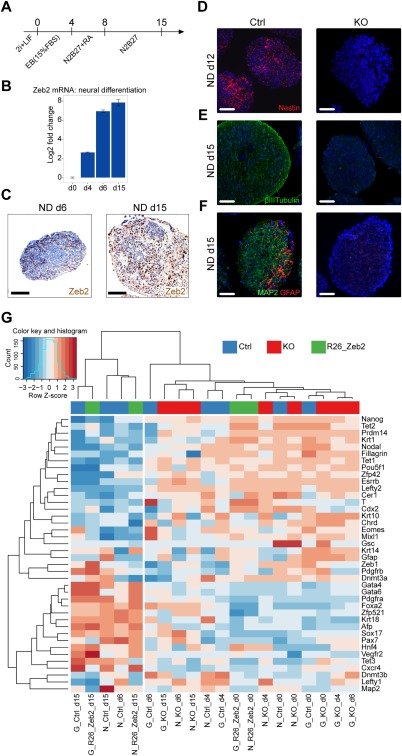
Knockout of *Zeb2* impairs embryonic stem cells (ESC) neural differentiation (ND) (for general differentiation [GD], see Supporting Information Fig. S1)**. (A)**: Scheme of the 15‐day ND protocol; RA: retinoic acid. **(B)**: RT‐qPCR of Zeb2 in Ctrl ESCs during ND. SD of two technical replicates is shown. **(C)**: Immunohistochemistry for Zeb2 (brown) in Ctrl embryoid bodies (EBs) (Ctrl) on d6 and d15 of ND. **(D‐F)**: Ctrl and Zeb2 KO (KO) ND‐EBs stained for Nestin (red, panel D) on d12, βIIITubulin (green, panel E) on d15 and costained for MAP2 (green) and GFAP (red) on d15 (panel F). Scale bars: 50 µm. Results shown are from one experiment and are representative for three experiments. **(G)** Heatmap for samples collected in pluripotency and during ND and GD with clustering based on Spearman correlation distances of quantile‐normalized RT‐qPCR values. Abbreviation: ND, neural differentiation.

EBs were fixed overnight with 4% paraformaldehyde followed by progressive alcohol‐assisted dehydration and paraffin embedding. 6‐μm sections were used for Immunohistochemistry (IHC) and Immunofluorescence (IF), which were carried out on Ventana *Ultra Discovery* (Roche, Vilvoorde, BE, www.ventana.com). The following antibodies were used: Zeb2 (custom antibody; Seuntjens et al., 27), βIIITubulin (Abcam, ab78078), Oct4 (Abcam, ab19857, Cambridge, UK, www.abcam.com), Nanog (Abcam, 80892), Cdh1 (BD Transduction Labs, 610182, Erembodegem, BE, www.bd.com), Tet1 (Millipore, 09‐872), Desmin (Abcam, ab8592‐500), Hnf4a (Abcam, ab41898), Sox17 (R&D Systems, AF1924, Abingdon, UK, www.rndsystems.com), and Alexa Fluor tagged secondary antibodies (Jackson Immunoresearch; 1:1,000, via Bio‐Connect, Huissen, NL, www.jacksonimmuno.com). For Figure 1A, ESCs were fixed for 10 minutes with ice‐cold paraformaldehyde and blocked for 30 minutes at 24°C with 0.1% Triton X100‐1% BSA in PBS. Anti‐Oct4 (Abcam, ab19857) and anti‐Nanog (Abcam, 80892) (both 1:1,000) were used as antibodies, with DAPI as nuclear counterstain (Life Technologies, D1306).

For the quantifications presented in Supporting Information Figure S7 (for Oct4, Hnf4a, Sox17, Tet1, and βIIITubulin), we manually quantified (using Fiji software) the number of positive cells as well as total numbers of DAPI+ cells and show the results as percentage (of +cells/total) instead of showing absolute cell numbers, because embryoid bodies have varying sizes. For the two non‐nuclear markers, E‐cadherin and Desmin, we used Fiji software to calculate the total area of staining and we normalized it to the total area of DAPI staining. We made the graphs and did the statistical analysis using Prism7 software.

### High‐Throughput Real‐Time PCR

In a first step non or unreliably expressed genes were removed based on quality information and a minimum threshold of 50% detection in all samples. Next, low quality samples were removed based on outlier detection of aggregation scores of all assay expression probabilities, calculated in all samples. Subsequently Ct values of the samples were quantile normalized and possible missing values were imputed using expression information of biological replicates. An overall limit‐of‐detection (LOD) was determined as the sum of the 75% quantile of normalized Ct values and a constant, that is, 10. To compare between assay levels and display on the graphs we retrieved log2 expression values by subtracting the LOD score with normalized Ct values and obtained rough absolute expression estimations by raising 2 to the power of the log2 score.

### RNA‐Seq Analysis

Total RNA was isolated using a Qiagen RNeasy (Qiagen, 74104, Antwerp, BE, www.qiagen.com). cDNAs were generated with Truseq RNA kit and sequenced (Illumina TruSeq v3 protocol on HiSeq2000, with a single read 36 bp and 7 bp index). Sequenced fragments were mapped to the mouse genome GRCm38 (Ensembl) using Tophat2 (v2.0.13). A count table for annotated genes was generated with featureCounts (v1.4.6); genes were further classified in different biotypes based on Vega gene and transcript annotation (vega.sanger.ac.uk/info/about/gene_and_transcript_types.html). *RNA‐seq expression data*: to compare counts between samples we converted them to Transcript Per Million (TPM) values. To retain only informative genes we filtered based on biotype, expression and standard variability using the aforementioned TPM values. First, we removed all genes belonging to short noncoding categories, in the next step we selected only these genes that have at least five transcripts/million in at least three samples and, finally, we removed the 20% lowest variable genes. The raw counts were imported in the R‐Package DESeq2 47 to test for differential expression between pairwise time‐points of KO and Ctrl samples or to perform time‐series analysis, therefore we created a design matrix that controls for differences at d0 and allows to assess the effect of factor time on gene expression between KO and Ctrl samples. *RNA‐seq clustering*: we used Principal Component Analysis (PCA) or unsupervised hierarchical clustering based on 1—Spearman correlation distance scores with average linkage. *RNA‐seq gene ontology*: to identify biological processes that are negatively enriched in *Zeb2* KO, we sorted genes according to their pi‐value (−log 10[*q* value] * log FC) based on DESeq2 time‐series analysis. The obtained ranked list was input for the GseaPreranked tool with only *–nperm 3,000, ‐set_max 500 ‐set_min 10* deviating from the default parameters. *RNA‐seq motif sequence analysis*: for imple motif analysis between KO and Ctrl at d6 we defined promoter regions as ± 2 Kb from the transcription start site (TSS) and counted the occurrences for putative binding site of Zeb2 (double YACCTG sequences with maximum gap of 40 bp) for all (up and down) differentially expressed genes (DEGs) (*p* < 0.01 and absolute log 2 FC > 1) and, as background, the promoter regions of all genes. One‐sided Fisher's exact test was used to determine significant over or under representation of this motif in promoter regions of DEGs relative to the genome‐wide promoter regions.

*Data deposition:* the RNA‐seq data have been deposited as data set GSE75618 and are available at www.ncbi.nlm.nih.gov/geo/query/acc.cgi?acc=GSE75618.

### DNA‐Methylome Analysis by RRBS

Total DNA was isolated by digestion with proteinase K and precipitation with isopropanol. RRBS was performed by NXT‐Dx (www.nxt-dx.com) using the premium RRBS kit (Diagenode). *RRBS processing*: the quality of sequencing reads was assessed by FastQC (v0.11.3_devel) and Trim Galore (v0.3.7) in *–rrbs* mode. These reads were then mapped to genome GRCm38 (Ensembl) using bismark (v0.14.1) with parameters *–bowtie2 –maxins* 1,000, allowing a maximum insert size of 1,000 bp for paired‐end sequences. To extract methylation information in a CpG context from both strands we used bismark_methylation_extractor with parameters *–paired‐end –no_overlap –comprehensive*. We used the R‐package methylKit 48 and custom R‐scripts to further analyze the data. In brief, we considered only CpGs with a minimum sequencing depth of 5x and removed the top 0.1% with highest coverage. To visualize global percentage methylation, histograms were created with 5%‐methylation bins. For all further analyses, we only retained CpGs that were present in all samples. *RRBS genomic regions*: genomic coordinates for genes were retrieved from GRCm38 and only coordinates for protein‐coding genes were used. We downloaded mm9 enhancer coordinates provided at http://chromosome.sdsc.edu/mouse/download.html, converted them to mm10 coordinates using CrossMap (v0.1.8), and extended them in both directions with 1 kb. CpG islands (CGI) and transposable elements (TE) were retrieved via the UCSC table browser for GRCm38/mm10, with the CGI and RepeatMasker tracks, respectively. The genomic coordinates for Canyons were retrieved from 49. We used a CpG observed/expected ratio of 0.29 to distinguish low and high‐CpG density promoters 50. Regions that do not belong to any of the aforementioned regions (e.g., intergenic regions) are described as “other.” *RRBS data analysis*: to identify differentially methylated regions (DMRs) and analyze global methylation dynamics/differences we averaged methylation in 400bp‐tiles containing at least three CpGs. Tiles with more than 20% difference in methylation and a *q* value <0.05 were assigned as significant DMRs, or simply DMRs.

*Data deposition:* the methylome analysis data have been deposited as a data subset of GSE75618 and are available at www.ncbi.nlm.nih.gov/geo/query/acc.cgi?acc=GSE75618.

### Analysis of Published Tet1‐Binding Peaks in ESCs

Data for Tet1 ChIP‐seq for mouse ESCs was downloaded from GEO (GSM659803, GSM659799). Reads were aligned to GRCm38 using bowtie with parameters *–e 70 –k 1 –m 1 –n 2 –concise*. Peaks were indicated with MACS software using default parameters. To study enrichment of Tet1 at demethylated regions, peaks were assigned to the closest demethylated region.

## Results

### ESC Differentiation is Impaired in Absence of Zeb2

We generated *Zeb2* KO 26, 39 along with *Zeb2^flox/flox^* control mESCs (Ctrl). In 2i + LIF, these ESC lines as population maintain high Nanog and Oct4 (Supporting Information Fig. S1A), proliferate comparably (Supporting Information Fig. S1B) and have a high and similar clonogenic capacity (±70%, *not shown*), showing that Zeb2 is dispensable for pluripotency and self‐renewal in ground‐state conditions.

Because of the documented role of Zeb2 in neural development 22, 23, 24, 27, 28, 29 we investigated ND of *Zeb2* KO ESCs, subjecting them as EBs to ND using retinoic acid [modified from Ref. 51] (Fig. 1A). In Ctrl EBs, the very low Zeb2 mRNA levels increased between day d0 and d4 after withdrawal of 2i + LIF as well as during the acquisition of neural fate (between d4 and d6) and remained high till the end of our 15‐day ND protocol (Fig. 1B). The first Zeb2‐positive (Zeb2+) cells are detected by IHC on d6, being intense from d8 (*not shown*) till the end of the experiment (Fig. 1C). Absence of neural progenitor (Nestin+), neuronal (βIIITubulin+, Map2+), and astroglial (GFAP+) markers (IF; Fig. 1D‐1F; for quantifications of neural conversion for the ESC lines discussed here and for other lines, see Supporting Information Fig. S7, here panel C) showed that ND was abolished in *Zeb2* KO EBs. Thus, Zeb2 is crucial for mESCs to acquire neural fate, in line with observations that Zeb2 KD in hESCs makes these cells favor mesendoderm over neuroectoderm fate 33.

To validate whether *Zeb2* genetic inactivation of in mESCs would also yield increase in mesendoderm, we subjected *Zeb2* KO ESC to general differentiation (GD; Supporting Information Fig. S1C) allowing commitment to all cell fates for 15d, and monitored Zeb2 mRNA/protein in Ctrl cells (Supporting Information Fig. S1D, 1E) and stained for mesoderm, endoderm, and neural markers, respectively (Supporting Information Figs. S1F‐1H, S7E‐7G). This showed that *Zeb2* KO mESC have impaired early differentiation not restricted to ND, but which affects all three germ layers.

Gene expression changes in *Zeb2* KO mESCs after exposure to differentiating cues were also analyzed via 40 marker mRNAs for neuroectoderm, mesoderm, endoderm and pluripotency, respectively, using reverse transcription quantitative polymerase chain reaction (RT‐PCR) on d0, 4, 6, and 15 in Ctrl and *Zeb2* KO cells, in ND and GD. Importantly, Zeb2 “rescue” ESC lines were included in this RT‐qPCR analysis (d0 and d15; Supporting Information Fig. S2F, 2G) by introducing Zeb2 (N‐tagged with Flag_3_/Strep‐tag) as cDNA in *R26* (see Supporting Information Experimental Procedures) of *Zeb2* KO cells (hereafter named R26_Zeb2). This restored the differentiation of these ESCs (IHC/IF, RT‐qPCR, see Supporting Information Figs. S2A‐2E, S7C, 7E‐7G). The expression heatmap (Fig. 1G) with samples clustered based on quantile‐normalized expression values showed clear separations between d15 Ctrl and R26_Zeb2 cells both in GD and ND, the d6 Ctrl in ND, and the rest of the samples including d15 *Zeb2* KO cells, further supporting our observation that *Zeb2* KO ESCs stay largely uncommitted and display overall reduced differentiation capacity.

### Zeb2 Acts Preferentially as a Transcriptional Repressor Associated with Developmental Progression

**Figure 2:**
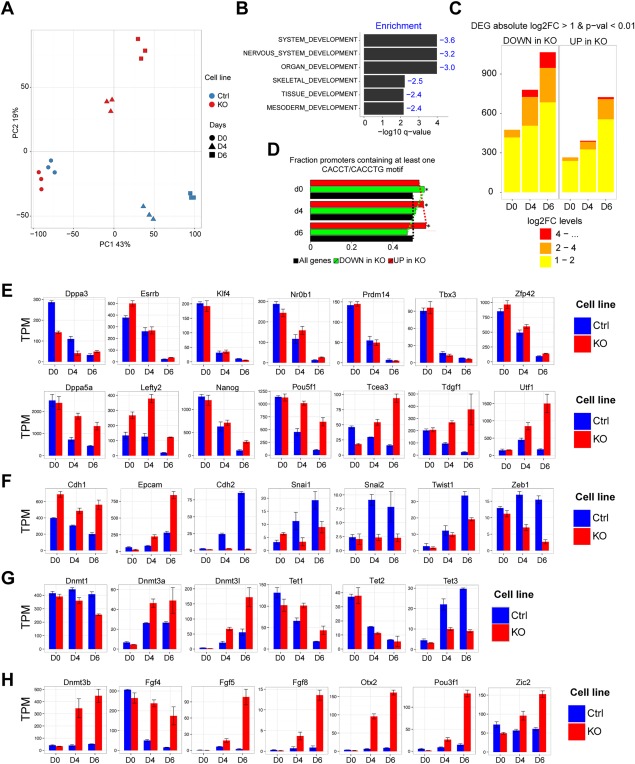
Analysis of temporal RNA‐seq. **(A)**: Principal component analysis based on transcripts per million (TPM). **(B)**: GSEA‐P for differentially expressed genes (DEG) in time‐series analysis. The height of the bar plot represents significance and the corresponding negative enrichment score is indicated (blue). **(C)**: Bar plot displays numbers of DEGs using pairwise DESeq2 test (|log 2FC| > 1 and *p* < 0.01). Colors represent binned absolute log2FC levels. **(D)**: Promoter analysis for putative bipartite Zeb2‐binding motifs (CACCT/CACCTG sequences with maximum gap of 45 bp; see main text) of DEGs between *Zeb2* KO versus Ctrl. Red bar = selective analysis for upregulated DEG, demonstrates statistical overrepresentation (Fisher's exact test *p* value = 1.044e‐08). Green bar = selective analysis for downregulated DEG points to underrepresentation. **(E‐H)**: TPM bar plots at the indicated time points for pluripotency‐related genes (E), selected epithelial‐to‐mesenchymal transition genes (F) and selected methylation‐related genes (G) and epiblast (H). Abbreviations: DEG, differentially expressed genes; TPM, transcripts per million.

Temporal RNA‐seq of Ctrl and *Zeb2* KO ESCs would show in more detail Zeb2‐dependent effects on early cell‐state/fate decisions and identified potential mediators of the impaired differentiation phenotype downstream of Zeb2. Here we chose ND wherein we can distinguish three stages that correspond in Ctrl cells to (i) ground‐state ESCs (d0, very low Zeb2 mRNA/protein), (ii) multipotent progenitors (d4, low Zeb2, cultured in presence of serum, induction of markers of three lineages are observed) (for details, see Supporting Information Fig. S3A‐3C; trophectoderm markers are documented in Supporting Information Fig. S3D), and (iii) early neural progenitors (d6, high Zeb2). For each stage we performed RNA‐seq for three independent experiments. PCA illustrated that both Ctrl and *Zeb2* KO on d0 are situated close together, but on d4 they already follow different trajectories (Fig. 2A). This coincides with the first induction of *Zeb2* (between d0 and d4 in Ctrl; Fig. 1B), indicating that Zeb2 influences cell‐fate decisions very early‐on when cells normally exit from their ground‐state and undergo lineage priming.

To gain insight into what processes might be involved in the establishment of the early differences between Ctrl and *Zeb2* KO cells, we identified the top positive and negative genes that contribute to principal component 2 (PC2, which separates the lineage trajectories of Ctrl and *Zeb2* KO samples on the PCA plot) and performed gene ontology (GO) analysis using Gorilla software 52. This revealed that the top‐100 genes associated with *Zeb2* KO cells were enriched for terms that relate to peptide biosynthesis and metabolism, while the top‐100 genes associated with Ctrl cells were mainly enriched for epigenome‐related terms, such as histone modification and chromatin organization (for the gene lists, see Supporting Information Table SIII).

We next applied a time‐series analysis (see Materials and Methods) on our RNA‐seq data set to assess the effect of the factor “time” and identify genes that have a different dynamic expression profile in KO versus Ctrl cells (Supporting Information Table SIV, RNASeq_Time_Series). Gene set enrichment (GSEA) analysis 53 of genes displaying this different dynamic behavior showed strong negative enrichment for various differentiation/developmental categories within the top‐10 hits (Fig. 2B) and further confirmed that at least the vast majority of *Zeb2* KO cells in EBs indeed remain uncommitted. We also (re‐)confirmed that *Zeb2* KO cells do not acquire neural fate (using *Pax6*, *Zfp521,* and *Neurog1*; Supporting Information Fig. S3A). The early‐neuroectoderm markers *Gbx2* and *Hoxa1* previously shown to be correctly induced upon differentiation in Zeb2 KD hESCs 33, were not induced in *Zeb2* KO mESCs. This indicates that genetic inactivation of *Zeb2* results in a more severe neural acquisition phenotype than the KD (Supporting Information Fig. S3A). We examined the expression of other cell lineage markers in our RNA‐seq data to exclude that *Zeb2* KO cells would preferentially induce non‐neural fates (Supporting Information Fig. S3B‐3D). Although a small increase in those markers was observed in Ctrl EBs, they were either almost absent (for mesoderm, Supporting Information Fig. S3C) or markedly lower (trophectoderm and endoderm; Supporting Information Fig. S3B, 3D) in Zeb2‐deficient EBs.

While this RNA‐seq data analysis significantly expands our previous characterization of the cells and confirms that *Zeb2* inactivation globally affects ESC differentiation, it also provided the possibility to discover potential Zeb2‐dependent candidate genes responsible for the impaired differentiation of *Zeb2* KO ESCs. Therefore, we performed pairwise RNA‐seq analysis at all three time‐points and identified DEGs (*p* value <0.01 and log 2 Fold Change (FC) >1; Supporting Information Table SV, RNAseq_Pairwise). Consistent with the divergent PCA trajectories we observed an increase in both number of DEGs and their FC over time (Fig. 2C).

Upon neural induction the majority of genes that were either up or down on d4 (multipotent progenitor stage) maintained this status on d6 (early neural progenitor), 69% and 72%, respectively. Numbers of DEGs increased between d4 and d6. To further filter for direct transcriptional regulation by Zeb2 we performed binding motif analysis within promoters (2 kb up and downstream of the transcription start site, TSS) of DEGs. We searched for two motifs, the E‐box sequence 5′‐CACCTG‐3′ and 5′‐CACCT‐3′, interspaced by 45 bp max 54. The genes upregulated during differentiation in *Zeb2* KO cells showed an increase in enrichment for the selected Zeb2‐binding motifs (Fig. 2D, red bars and line), while the opposite trend was observed for downregulated genes (Fig. 2D, green bars and line). This suggests that Zeb2 functions preferentially as a transcriptional repressor during differentiation.

### *Zeb2* KO ESCs Stall in an Epiblast‐like State

Zeb2‐deficiency leads to impaired differentiation of ESCs and Zeb2 preferentially acts as repressor. We, therefore, investigated whether the pluripotency network was properly silenced in *Zeb2* KO ESCs, in particular the genes associated with the naïve state and known as rapidly downregulated upon withdrawal of 2i + LIF 38. *Klf4*, *Tbx3*, *Zfp42*, *Prdm14*, *Essrb, Nr0b1,* and *Dppa3* were all properly downregulated in both *Zeb2* KO and Ctrl ESCs (Fig. 2E, upper panel). However, a significant set of factors that are part of a larger pluripotency network or involved in initiation of differentiation were not at all or only partially downregulated, such as *Lefty2*, *Tcea3*, *Dppa5a*, *Utf1,* and *Tdgf1* 44, 45 (Fig. 2E, lower panel). This group also included *Pou5f1* and *Nanog*, key players in the acquisition of pluripotency and early development 55, 56. All genes in the latter group contain putative binding sites for Zeb2 within 2 kb from their TSS, suggesting that Zeb2 is a candidate direct repressor of (at least some) genes involved in pluripotency maintenance. In line with the role of Zeb2 in EMT 19 we observed that in *Zeb2* KO cells *Cdh1*expression remains high, *Epcam* is strongly induced and *Cdh2*, *Snai‐1*/*2*, *Twist1,* and *Zeb1* were not induced to the same extent in differentiation conditions (Fig. 2F). This confirms that these ESCs have defective EMT consistent with previously documented roles of Zeb2, including downregulation of *Cdh1*, in other cell types.

Both *Dnmt3b* (Fig. 2H) and *Dmt3l* (Fig. 2G) have putative Zeb2‐binding sites and were upregulated in *Zeb2* KO during differentiation. Together with other genes they determine DNA‐methylation at this stage, hence we monitored *Dnmt1*, *Dnmt3a, Tet1*, *Tet2,* and *Tet3* expression 10, 12, 14, 15. In addition to high expression of all three *Dnmt3* genes, the *Tet1/2* to *Tet3* expression switch is only partially achieved; it normally occurs during transition from pluripotent stem cells to differentiated cells 15, but in our case *Tet3* induction is limited and *Tet1* expression is higher (Fig. 2G).

Although EBs are inherently heterogeneous, all our aforementioned results indicate that at least part of the cells are stalled in an epiblast‐like cell state in which early epiblast markers are induced whereas a number of pluripotency genes gets downregulated 45, 56, 57. We observed a strong increase of expression of the established postimplantation epiblast genes *Otx2*, *Pou3f1 (Oct6)*, *Dnmt3b, Zic2,* and *Fgf5* 53, 58 (Fig. 2H). This data suggests that at least a fraction of *Zeb2* KO cells undergoes lineage priming and acquires epiblast‐like cell (EpiLC) features.

### *Zeb2* KO ESCs Display Less Efficient ESC‐to‐EpiLC Conversion

**Figure 3:**
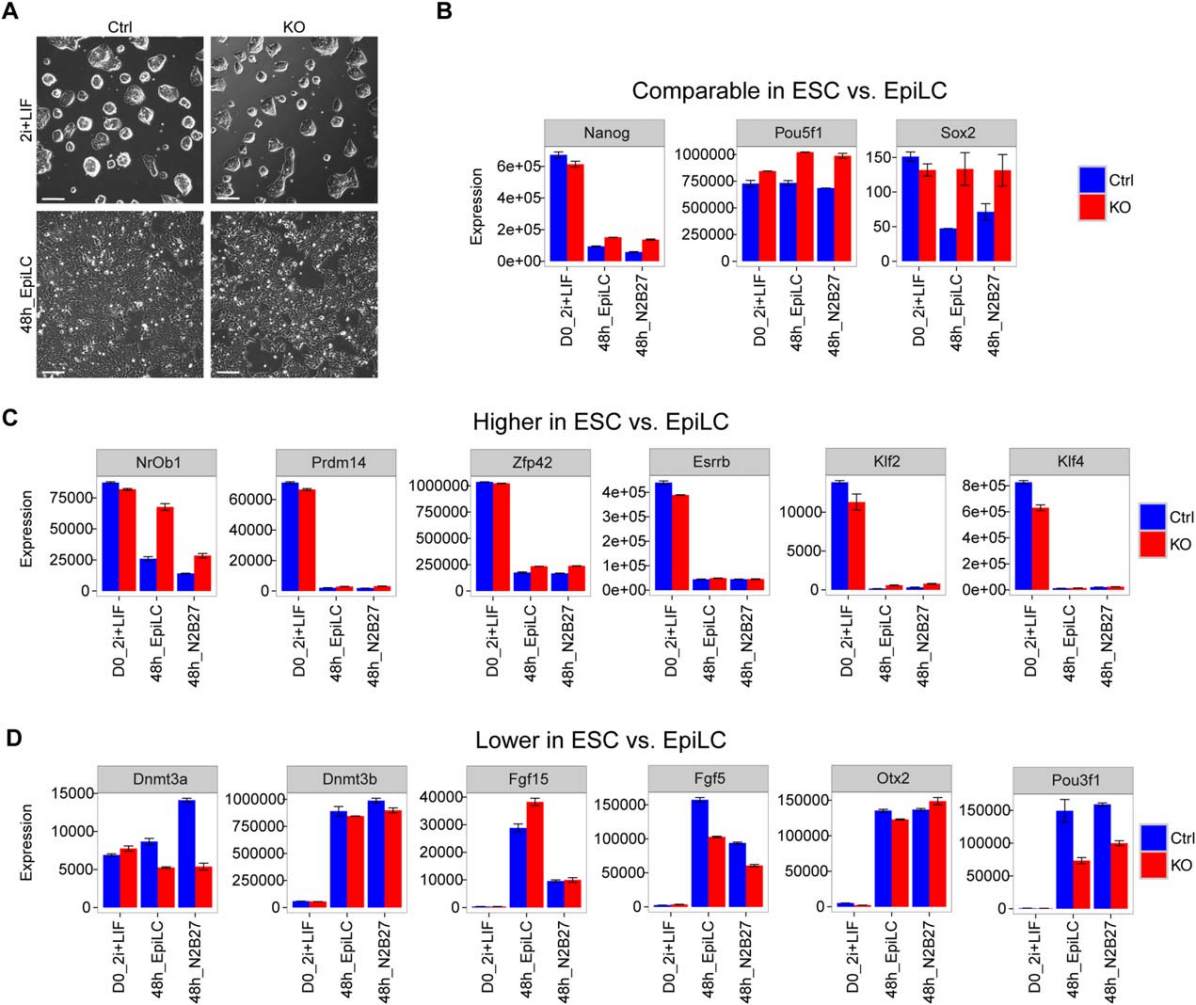
*Zeb2* KO embryonic stem cells (ESCs) display less efficient ESC‐to‐EpiLC conversion. **(A)**: Phase‐contrast images of Ctrl and Zeb2 KO cells in 2i + LIF and after 48 hours of EpiLC conversion. Scale bar: 50 μM. **(B‐D)**: RT‐qPCR analysis of Ctrl and *Zeb2* KO samples on d0, after 48 hours in EpiLC medium and after 48 hours N2B27 in medium. (B): Transcripts comparable between ESC and EpiLC (Nanog, Pou5f1, Sox2). **(**C): Transcripts higher in ESC versus EpiLC (Nr0b1, Prdm14, Zfp42, Esrrb, Klf2, Klf4). (D): Transcripts lower in ESCs versus EpiLCs (Dnmt3a, Dnmt3b, Fgf15, Fgf5, Otx2, Pou3f1). These three categories (shown in panels B, C, D) are based on 66. Results shown are from one experiment; error bars are from three biological samples. Abbreviation: ESCs, embryonic stem cells.

To test whether *Zeb2* KO cells could acquire to epiblast‐like fate, we subjected them (in parallel with the Ctrl line) to a 48 hour‐long ESC‐to‐EpiLC conversion 45 and examined the transcriptional changes, using high‐throughput qPCR (see Materials and Methods), of a set of markers shown to be comparably and differentially expressed, respectively, between ESCs and EpiLCs [based on Ref. 55]. To evaluate whether EpiLC acquisition depends on the presence of Fgf2 + ActivinA, we included also samples of 48 hour‐long differentiation in pure N2B27. There were no obvious morphological differences between Ctrl and *Zeb2* KO cells in 2i + LIF or after 48 hours ESC‐to‐EpiLC conversion and both lines acquired more flat morphology (Fig. 3A).

First, we analyzed expression of *Nanog*, *Oct4* and *Sox2*, which according to Buecker and coworkers are comparable between ESCs and EpiLCs 57. *Nanog* was downregulated in both Ctrl and *Zeb2* KO lines after 48 hours, but in the *Zeb2* KO cells it was still expressed at higher levels (for *Nanog* downregulation, see 45). *Oct4* was retained in Ctrl cells and slightly increased in the *Zeb2* KO cells after 48 hours, while *Sox2* was downregulated and retained, respectively (Fig. 3B). Next, we looked at *Nr0b1*, *Prdm14*, *Zfp42*, *Esrrb*, *Klf2,* and *Klf4*, which should be expressed at higher level in ESCs as compared to EpiLCs. All these markers were found downregulated in both lines, but in the *Zeb2* KO line *Nr0b1*, *Zfp42,* and *Klf2* continued to display higher mRNA levels as compared to Ctrl cells, whereas *Prdm14*, *Esrrb,* and *Klf4* mRNA reached after 48 hours similar levels in both lines (Fig. 3C). Last, we analyzed *Dnmt3a*, *Dnmt3b*, *Fgf15*, *Fgf5*, *Otx2,* and *Pou3f1*, a set of markers expected to be expressed at lower levels in ESCs as compared to EpiLCs. With the exception of *Dnmt3a* in the *Zeb2* KO line, all markers were induced in both Ctrl and KO cell lines. In particular, *Dnmt3b* and *Otx2* are induced in Ctrl and *Zeb2* KO lines to the same extent, while *Fgf5* and *Pou3f1* show higher mRNA levels in Ctrl versus *Zeb2* KO cells. *Fgf15* in EpiLC conversion is expressed at higher levels in the *Zeb2* KO line as compared to Ctrl and after 48 hours in N2B27 its expression is comparable in both lines (Fig. 3D). Taken together, the results obtained in ESC‐to‐EpiLC conversion and 48 hours of N2B27 are comparable, meaning that the transcriptional changes of genes analyzed are not influenced by the presence of Fgf2 + ActivinA in the medium.

We conclude that, at population level, *Zeb2* KO cells present with a less efficient conversion to EpiLC phenotype, likely resulting from a combination of both naïve and primed states. Since epiblast fate requires more efficient pluripotency gene silencing than observed in the *Zeb2* KO cells and a significant induction of markers such as *Fgf5* and *Pou3f1*, we suggest that—in our EBs—a fraction of *Zeb2* KO cells remains in naïve ESC state, while the remaining cells may still undergo EpiLC conversion. Single‐cell mRNA analysis is needed in future experiments to provide insight into the proportions of cells that stay in the naïve versus primed state as well as revealing the presence of (few) cells progressing toward differentiation.

### Pluripotent Potential Is Retained in *Zeb2*‐Deficient Embryoid Bodies

**Figure 4:**
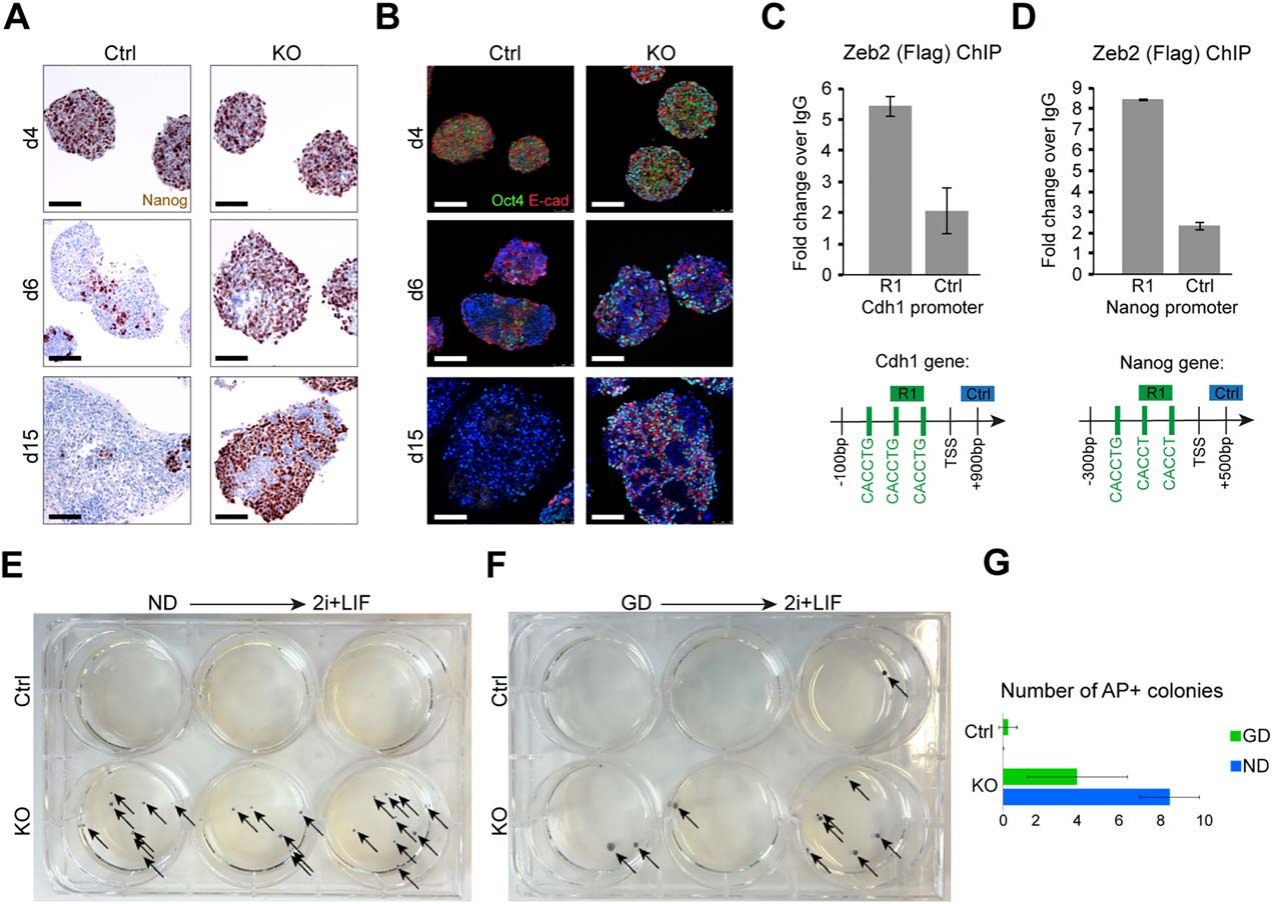
Pluripotency genes are not efficiently downregulated during differentiation in *Zeb2* knockout (KO) embryonic stem cells (ESCs). **(A)**: Control (Ctrl) and *Zeb2* KO (KO) embryoid bodies (EBs) stained for Nanog (brown) on d4, d6, and d15 of neural differentiation (ND). **(B)**: Ctrl and KO EBs costained for Oct4 (green) and Cdh1 (red) on d4, 6, and 15 of ND. Panels A, B show results from one experiment that is representative for three experiments. Scale bar: 50 µm. **(C, D)**: Zeb2 chromatin immunoprecipitation (using anti‐Flag antibody) on *Cdh1* (panel C) and *Nanog* promoter (panel D). Results shown are from one experiment and representative for three experiments. SD of two technical repeats is shown. **(E, F)**: Ctrl and KO ESCs subjected to ND (panel E) and general differentiation (panel F) for 15 days, dissociated and plated at 500 cells/well in 2i. The resulting ESC colonies (indicated by arrows) were visualized by staining for AP, and panel **(G)** represents the average number of AP+ colonies obtained after plating the cells. Abbreviations: ND, neural differentiation; GD, general differentiation.

Pou5f1 (Oct4) and Nanog, two crucial pluripotency‐supporting factors maintained in epiblast cells, remained high (as seen by western blotting and RT‐qPCR, Supporting Information Fig. S5A, 5B) and were present in a large fraction of cells in *Zeb2* KO ND‐EBs till d15 (Fig. 4A, 4B). Again, this observation could be extended to GD‐EBs (Supporting Information Fig. S5C, 5D; d15). In addition, high numbers of Cdh1+ cells were observed in *Zeb2* KO EBs (Fig. 4B) and this was also seen at protein and mRNA levels on d15 (Supporting Information Fig. S5A, 5B). R26_Zeb2 rescue partially restored downregulation of Oct4 (and Cdh1) mRNA/protein (Supporting Information Fig. S2C). To confirm that this is the direct result of Zeb2 binding a ChIP‐qPCR was carried out over the Zeb2‐binding motif (Fig. 4C, 4D). This showed enrichment of Flag‐tagged Zeb2 (using R26_Zeb2 ESCs) *Cdh1* promoter 19, 26 and its new candidate target *Nanog*.

To document the persistence of the pluripotent state upon differentiation in *Zeb2* KO cells, we dissociated Ctrl and *Zeb2* KO EBs on d15 (in ND or GD), sorted the living cells and plated these at 500 cells/well as single cells in 2i + LIF. Alkaline phosphatase (AP)+ ESC colonies derived from EBs subjected to differentiation (Fig. 4E, 4F) were quantified on d9 (Fig. 4G). In a typical experiment, Ctrl cells subjected to ND did not give rise to AP+ cells, whereas *Zeb2* KO cells in 2i + LIF yielded on average 8 colonies/well. In GD, Ctrl cells gave rise to less than 1 (calculated 0.2) AP+ colony/well, whereas for *Zeb2* KO cells this was 4 colonies/well on average. Based on AP read‐out, this shows these latter cells have the remarkable ability to re‐adapt to 2i + LIF, like ESCs and EpiSCs, and that they form AP+ colonies even up to d15 of differentiation treatment. Without assessment at single‐cell level, we cannot discriminate whether these AP+ colonies arose exclusively from epiblast‐like or more naïve *Zeb2* KO cells since both cell types can adapt to 2i + LIF. Teratoma formation, using EBs subjected to ND for 12 days showed that Ctrl EBs failed to form teratomas, while *Zeb2* KO EBs gave rise to teratomas in 4 weeks (Supporting Information Fig. S5E). These data show, therefore, that *Zeb2* genetic inactivation leads to maintenance of pluripotency even after prolonged differentiation.

### The *Zeb2* KO Embryoid Bodies, Subjected to ND, Fail to Maintain the Initially Acquired DNA‐Methylation

**Figure 5:**
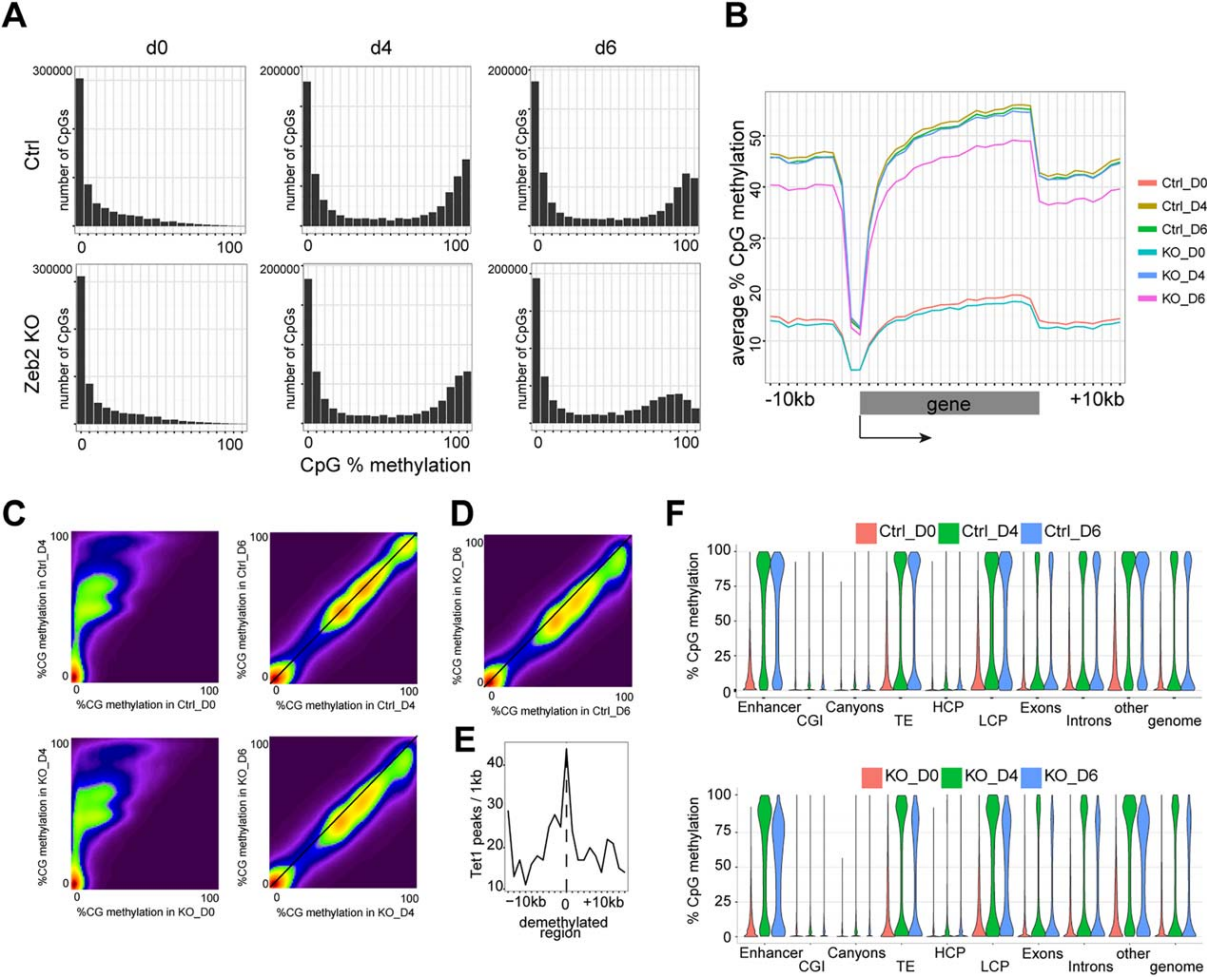
Analysis of temporal RRBS during neural differentiation. **(A)**: Distribution histogram for individual ^me^CpGs on d0, d4, and d6 in Ctrl and *Zeb2* KO populations. **(B)**: ^me^CpG distribution at gene bodies and 10kb‐flanking regions of protein‐coding genes. **(C)**: Density plots for pairwise comparisons of ^me^CpG (in 400bp‐tiles) between d0 and d4 in Ctrl (top) and d4 and d6 in KO (bottom) cells. **(D)**: Density plot for pairwise comparison of ^me^CpG (in 400bp‐tiles) on d6 between Ctrl and KO. In C, D the density points increase from purple to dark red. **(E)**: Enrichment plot of Tet1‐binding peaks centered around demethylated regions on d6 in a pairwise comparison between *Zeb2* KO versus Ctrl. **(F)**: Violin plots showing gain and loss‐of‐methylation over time in identified genomic regions, that is, enhancers, CpG islands, canyons, transposable elements, high‐CpG content promoters (HCP), low‐CpG content promoter, exons, introns, other nondefined genomic regions, and globally at the whole‐genome (genome) in Ctrl and *Zeb2* KO cells. Abbreviations: CGI, CpG islands; TE, transposable elements; HCP, high‐CpG content promoters; LCP, low‐CpG content promoter.

*Zeb2* KO EBs show impaired cell differentiation and deregulated expression of the core methylation machinery genes. This prompted us to examine the acquisition and maintenance of CpG‐methylation (^me^CpG) that accompanies the decision of irreversible ESC differentiation. Retaining the same time/sample setups as for RNA‐seq and again using ND, single‐base profiles were generated of methylation by RRBS in both Ctrl and *Zeb2* KO on d0, d4, and d6. The genome of ground‐state (d0) ESCs was globally hypomethylated 59. On d4 both cell populations gained methylation in agreement with our observation that they are in an epiblast‐like (for KO) or multipotent (Ctrl) state. However a significant drop of ^me^CpG was observed in the d6 *Zeb2* KO cell population, suggesting that part of the CpG methylation is lost (Fig. 5A). The progressive accumulation of ^me^CpG in our EBs has a striking resemblance with that observed in vivo 60, that is, our d0 population profile is similar to blastocyst‐stage embryos (between E3.5 and E4.5), while d4 and d6 Ctrl populations have a similar distribution profile as epiblast embryos (E6.5). In contrast, KO d6 resembles early‐epiblast embryos (E5.5) with a reduction in ^me^CpG at both gene bodies and 10kb‐flanking regions (Fig. 5B) 60.

We further investigated changes in the (de)methylation process by considering CpGs covered in all samples and averaging methylation in 400bp‐tiles, with a total of 1,84,564 tiles. This identified differentially methylated regions (DMRs) (absolute methylation change >20% and *q* value <0.05) in both a time and pairwise‐dependent manner (Supporting Information Table SVI, RRBS_Pairwise). Both Ctrl and KO cells significantly gained methylation in respectively 33.8% and 33.5% of all tiles between d0 and d4 (Fig. 5C, left panels; Supporting Information Fig. S6A). During this period, no single significant loss‐of‐methylation was observed (Supporting Information Fig. S6B). Next, between d4 and d6 Ctrl cells maintained a very stable level of methylation with only little gain or loss‐of‐methylation, that is, 0.1% of all tiles (Fig. 5C, right top panel; Supporting Information Fig. S6A, 6B). In agreement with the observed overall lower methylation at d6 (Fig. 5A, 5B), KO cells had 10 times more tiles (1806 or 1% of all tiles) with significant loss‐of‐methylation and only 90 tiles (0.05% of all tiles) with gain‐of‐methylation (Fig. 5C, right bottom panel; Supporting Information Fig. S6A, 6B; see also Supporting Information Table SVII, RRBS_Temporal). Furthermore, analysis of these aforementioned DMR in both Ctrl and KO cells revealed that these regions initially acquired methylation in both Ctrl and KO cells at d4, but this methylation was only maintained in Ctrl cells (Supporting Information Fig. S6C).

To investigate whether demethylation was selective for specific genomic regions, we profiled the methylation dynamics of enhancers, CGI, canyons, TE, high‐CpG content (HCP) and low‐CpG content promoters (LCP), exons and introns. As reported before 49, resistance to gain‐of‐methylation occurs for canyons and high‐CpG regions (CGI and HCP), while all other regions (enhancers, TE, LCP, exons, and introns) were susceptible to gain‐of‐methylation. In contrast, the *Zeb2* KO population is unable to maintain this methylation initially acquired in all aforementioned genomic regions (Fig. 5F).

### Failure to Maintain Acquired DNA‐Methylation During ND Is Associated with Tet1‐Binding; Tet1 Knockdown in *Zeb2* KO ESCs Facilitates Silencing of *Nanog*, *Oct4,* and *Cdh1* and Partially Rescues the Lineage Differentiation Phenotypes

Regions that lose methylation in d6 *Zeb2* KO populations initially acquired methylation comparable to Ctrl (Supporting Information Fig. S6C). We also compared d6 of both Ctrl and *Zeb2* KO populations and as expected observed a similar number of tiles with loss‐of‐methylation (1938, or 1% of all tiles) (Fig. 5D) and we observed also an increased level of Tet1 (Fig. 2). We, therefore, asked whether the regions that lose methylation correlate with Tet1‐binding. Figure 5E shows that regions that lose methylation in *Zeb2* KO cells are enriched for Tet1‐binding in normal ESCs: we could do this by combining analysis of published ChIP‐seq data for Tet1 in mESCs 61 with our region‐specific loss of methylation data on d6 (compared between Ctrl and *Zeb2* KO). This strongly suggests that the observed demethylation in the *Zeb2* KO cells is an active process mediated by elevated Tet1 levels in agreement with DNA‐demethylation being initiated at Tet1‐binding sites 62.

**Figure 6:**
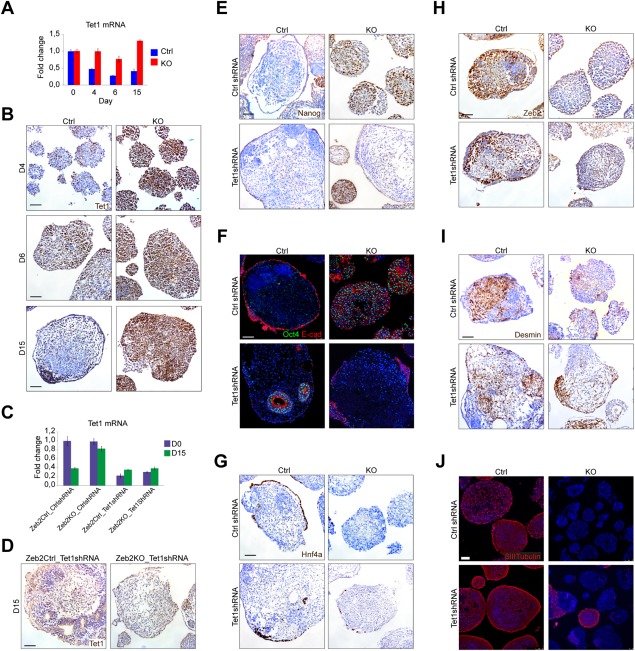
Tet1 knockdown in *Zeb2* knockout embryonic stem cells facilitates their definitive pluripotency exit and partially restores their neural (neural differentiation [ND]) and general differentiation (GD) defect. **(A)**: RT‐qPCR of Tet1 mRNA in Ctrl (blue) and *Zeb2* KO (red) lines on d0, d4, d6, and d15 of ND. SD of two technical replicates is shown. **(B)**: Ctrl and *Zeb2* KO embryoid bodies (EBs) stained for Tet1 (brown) on d4, d6, and d15 of GD. **(C)**: RT‐qPCR of Tet1 mRNA on d0 (violet) and d15 (green) in Ctrl_CtrlshRNA, Zeb2KO_CtrlshRNA, Ctrl_Tet1shRNA and Zeb2KO_Tet1shRNA lines. SD of two technical replicates is shown. **(D)**: Ctrl_Tet1shRNA and Zeb2KO_Tet1shRNA EBs costained for Tet1 (brown) on d15 of GD. Scale bar B, D: 50 µm. **(E‐J)**: Ctrl_CtrlshRNA, Zeb2KO_CtrlshRNA, Ctrl_Tet1shRNA and Zeb2KO_Tet1shRNA EBs stained for the indicated markers. (E): Nanog (brown) on 15 of GD. F. Oct4 (green) and Cdh1 (red) on d15 of GD. (G): Hnf4a (brown) on d15 of GD. (H): Zeb2 (brown) on d15 of GD. (I): Desmin (brown) on d15 of GD. (J): βIIITubulin (red) on d15 of ND. Scale bar: 75 µm. (E‐I): Scale bar: 50 µm. In all panels, results shown are from one experiment and representative for three experiments.

Tet1 remains high in the *Zeb2* KO EBs even on d15 of differentiation in contrast to its normal downregulation during ND and GD (Fig. 6A, 6B). To test whether high Tet1 levels lead to inefficient silencing of *Nanog*, *Oct4,* and *Cdh1* and hence a block in differentiation of these cells, we transduced control and *Zeb2* KO ESC lines with a lentivirus expressing shRNA directed against Tet1 (called Ctrl_Tet1shRNA, Zeb2KO_Tet1shRNA, respectively). Tet1 was almost undetectable in Ctrl and *Zeb2* KO lines targeted with Tet1 shRNA (Fig. 6C, 6D; for quantifications of Tet1 staining, see Supporting Information Fig. S7A). In 2i + LIF, the Tet1 KD lines maintained their undifferentiated characteristics (*not shown*). We subjected these Tet1shRNA lines to ND and GD, respectively, along with the same lines receiving control non‐targeting shRNA (Ctrl_CtrlshRNA, Zeb2KO_CtrlshRNA). These control shRNA lines behaved as expected in differentiation (Fig. 6E‐6J), and Zeb2 was indeed absent from Zeb2KO_CtrlshRNA and Zeb2KO_Tet1shRNA EBs at the end of GD (Fig. 6H) and ND (*data not shown*). After 15 days, Zeb2KO_Tet1shRNA cells subjected to either ND or GD efficiently decreased Nanog, Oct4, and Cdh1 mRNA to low levels at the end of GD (Fig. 6E, 6F; ND *data not shown*; for quantifications of Oct4 and Cdh1, see Supporting Information Fig. S7B, 7D). In Zeb2KO_Tet1shRNA lines subjected to GD, partial rescue of differentiation to mesoderm (Fig. 6I; for quantifications of Desmin, see Supporting Information Fig. S7G) and endoderm (Fig. 6G; for quantifications of Hnf4a and Sox17, see Supporting Information Fig. S7E, 7F) was observed, but not to neuroectoderm, (*data not shown*). Partial rescue of ND was observed only when Zeb2KO_Tet1shRNA cells were subjected to ND (Fig. 6J; for quantification of βIIITubulin, see Supporting Information Fig. S7C). Thus, Tet1 remains high in *Zeb2* KO cells during differentiation, and forced downregulation of Tet1 in these cells in such conditions enables decreasing *Nanog*, *Oct4,* and *Cdh1* transcription and partially rescues differentiation. We conclude that Zeb2‐deficiency during differentiation leads to higher Tet1, which is associated with improper reduction of *Nanog* and *Oct4,* resulting in impaired differentiation.

## Discussion

Using *Zeb2* genetic inactivation in ESCs for the first time as well as rescue in such *Zeb2* KO cells via reintroduction of *R26*‐driven Zeb2‐cDNA, Zeb2 is shown critical for these cells to undergo three‐lineage differentiation. We propose that Zeb2 drives lineage commitment and specification by acting on multiple sets of Zeb2‐dependent genes. First, Zeb2 is an important EMT‐inducer 19. *Zeb2* KO ESCs retain epithelial characteristics when subjected to differentiation. Their phenotype appears even more severe than the recently described KO in ESCs of another known EMT‐regulator, *Snai1*, which still differentiate 63. Second, the downregulation of important pluripotency network regulators depends on Zeb2. In contrast to Ctrl, *Zeb2* KO ESCs retain high Tet1, Oct4, and Nanog during differentiation. In ESCs, Tet1 is involved in a positive regulatory loop with Nanog and Oct4. Tet1 co‐operates with Nanog, while the KD of Nanog weakens Tet1‐binding to its targets (including *Oct4*, *Esrrb*). Tet1 was also shown to act downstream of Oct4, and downregulation of Oct4 leads to decreased Tet1 14, 15, 17, 18. Tet1 acts downstream of histone deacetylase Sirt6 to control ESC fate in differentiating conditions 50. Also, like in our system, the Tet1 KD allowed silencing of *Oct4* and *Nanog* and rescued the *Sirt6* KO differentiation defect. Thus, Tet1 has a global inhibitory role in regulating key pluripotency genes during ESC differentiation, and this work identifies Zeb2 as an (indirect) upstream factor important for achieving correct Tet1 levels.

We describe a link between Zeb2 and regulation of DNA‐methylation status. Acquisition of DNA‐methyl marks during embryogenesis is thought to be unidirectional 60, but studies in ground‐state naïve ESCs and EpiSC, respectively, show that the methylomes are interconvertible in vitro when different conditions are applied 59, 64. Our RRBS showed that correct DNA‐methylation patterns are initially acquired by *Zeb2* KO cells, but that this pattern cannot be sustained: *Zeb2* KO cells revert the methylome to a more naïve state, which agrees with the maintenance of their undifferentiated phenotype associated with persistence of Nanog and Oct4. Remarkably, this reversion in *Zeb2* KO cells is facilitated in absence of additional cues or signals, like LIF and/or 2i. We hypothesize that Tet1 levels are maintained by the key pluripotency genes in *Zeb2* KO cells. Steady‐state high‐Tet1 would then actively demethylate the genome and contribute to preserving high‐Nanog and high‐Oct4 in the mutant cells. Enrichment of Tet1‐binding at regions that lost methylation in *Zeb2* KO cells further supports this hypothesis. Tet1 KD in these *Zeb2* KO cells facilitated downregulation of *Nanog* and *Oct4* as well as *Cdh1*, but their differentiation phenotype was only partially rescued.

The aforementioned discussed results raise the question on how it is possible that *Zeb2* KO ESCs, in which Tet1 mRNA is not downregulated, can still undergo DNA‐methylation. First, the main function of Tet1 may be to actively catalyze demethylation rather than prevent methylation per se 12, 13. We hypothesize that between days 0 and 4 the gain‐of‐methylation in both Ctrl and Zeb2 KO cells is driven by the very early events linked to the withdrawal of 2i + LIF and entering the primed state of pluripotency. We have, however, not documented that what seems like an equal total gain in Ctrl and KO cells, also occurs with the same dynamics (because we analyzed in detail only days 0 and 4 of differentiation) and altogether reflects precisely that these cells undergo the same changes. In other words, Ctrl cells could be entering three‐lineage differentiation program(s) (which we were able to confirm by analyzing their transcriptional profile) whereas *Zeb2* KO cells could be stalled in the epiblast‐like state and both changes would manifest by the same gross methylation pattern. It has been previously published that there is a large gain of methyl marks in ESCs when they transit from ground (2i + LIF) to serum + LIF conditions, both of which maintain functional pluripotency 62. Hence, the observed acquisition of methyl marks can be partially a reflection of entering the metastable state by the Zeb2 KO cells.

It has previously been shown that *Dnmt1* KO ESCs show decreased total DNA‐methylation levels, whereas DNA of *Dnmt* ^+/−^ mutant ESCs is still highly methylated 65. It could be that the observed loss of DNA‐methylation in our *Zeb2* KO ESCs on d6 is partially caused by decreased Dnmt1 levels that cannot sustain the acquired methylation pattern in the presence of high Tet1, which continuously catalyzes DNA‐demethylation. Interestingly, the expression of *Dnmt3a/3b/3l* was higher in *Zeb2* KO as compared to Ctrl ESCs. We hypothesize that the observed increase in de novo methyltransferase gene expression could be a counter‐acting mechanism to sustain the balance between DNA‐methylation and demethylation. The end result, loss of DNA‐methylation, could hence be due to high constant levels of Tet1 that on itself is sustained by the pluripotency genes.

Using GO analysis (done on PC2), we also noted that aberrant chromatin changes and histone modifications could contribute to the differentiation phenotype in *Zeb2* KO ESCs. ESCs have a unique, open chromatin that changes rapidly upon cell differentiation, thereby influencing transcriptional regulation and cell identity 4, 62. Given the differences in the transcriptional profile of Ctrl versus *Zeb2* KO ESCs, we hypothesize that, in addition to the DNA‐methylation related phenotype followed‐up here, *Zeb2* KO ESCs might also have a different chromatin (more ESC‐like) structure, which contributes to their undifferentiated phenotype.

It is also likely that Zeb2 controls other important cell fate regulators at multiple stages of differentiation in addition to the pluripotency genes and *Tet1* investigated here. For example, similar to described in vivo functions of Zeb2 in myelinogenesis in embryonic CNS 32, Zeb2 may also counteract genes that are inhibitory for neural conversion during ESC differentiation; it may also act as an activator of other target genes depending on its cofactors 66, 67 which altogether would then promote neurogenesis. Subsequent work will have to encompass the mapping of the genome‐wide binding sites of Zeb2 in mESCs. As the current anti‐Zeb2 antibodies fail in such studies (*not shown*), these studies will require an endogenous tagging approach within *Zeb2* to identify Zeb2 DNA‐binding sites and also stage‐relevant protein partners of Zeb2.

## Conclusion

The transcription factor Zeb2 is critical for exit from the epiblast state in mouse ESCs and links the pluripotency network and DNA‐methylation with irreversible commitment to differentiation. *Zeb2* KO ESCs display impaired differentiation by stalling in an epiblast‐like state. Using RNA‐seq, we conclude that in differentiating conditions EMT, pluripotency, lineage commitment and DNA‐(de)methylation genes are deregulated in *Zeb2* KO embryoid bodies. Using RRBS, we demonstrate that these cells cannot maintain their initially acquired DNA‐methylation marks in neural‐stimulating condition and do not effectively downregulate *Oct4*, *Nanog,* and *Tet1* in differentiation conditions. Tet1 KD partially rescues the impaired differentiation of the KO cells.

## Author Contributions

A.S.: Conception and design, collection and/or assembly of data, data analysis and interpretation, manuscript writing, final approval of manuscript; R.D.: Conception and design, collection and/or assembly of data, data analysis and interpretation, manuscript writing, final approval of manuscript; T.P.: Conception and design, provision of study material or patients, collection and/or assembly of data, data analysis and interpretation, manuscript writing, final approval of manuscript; G.V.: Conception and design, provision of study material or patients; AC.: Conception and design, manuscript writing; K.C.: Collection and/or assembly of data; A.F.: Collection and/or assembly of data; L.U.: Collection and/or assembly of data; W.V.I.: Collection and/or assembly of data, final approval of manuscript; G.B.: Provision of study material or patients; L.v.G.: Conception and design, provision of study material or patients, final approval of manuscript; F.G.: Conception and design, manuscript writing, final approval of manuscript; S.G.: Conception and design, provision of study material or patients, collection and/or assembly of data, manuscript writing, final approval of manuscript; J.H.: Conception and design, financial support4. Provision of study material or patients, collection and/or assembly of data, manuscript writing, final approval of manuscript; D.H.: Conception and design, financial support, data analysis and interpretation, manuscript writing, final approval of manuscript.

## Disclosure of Potential Conflicts of Interest

The authors indicate no potential conflicts of interest.

## Supporting information

Supporting Information Figure 1

Supporting Information Figure 2

Supporting Information Figure 3

Supporting Information Figure 4

Supporting Information Figure 5

Supporting Information Figure 6

Supporting Information Figure 7

Supporting Information Tables 1‐2

Supporting Information Tables 2‐3

Supporting Information Table 3

Supporting Information Table 4

Supporting Information Table 5

Supporting Information Table 6

Supporting Information Table 7

Supporting Information Figure Caption
