# Supplementary figures and images for: Zeb2 Regulates Cell Fate at the Exit from Epiblast State in Mouse Embryonic Stem Cells
Source: Stem Cells. 2016 Nov 8;35(3):611–25. doi: 10.1002/stem.2521 (PMC5396376; doi:10.1002/stem.2521)

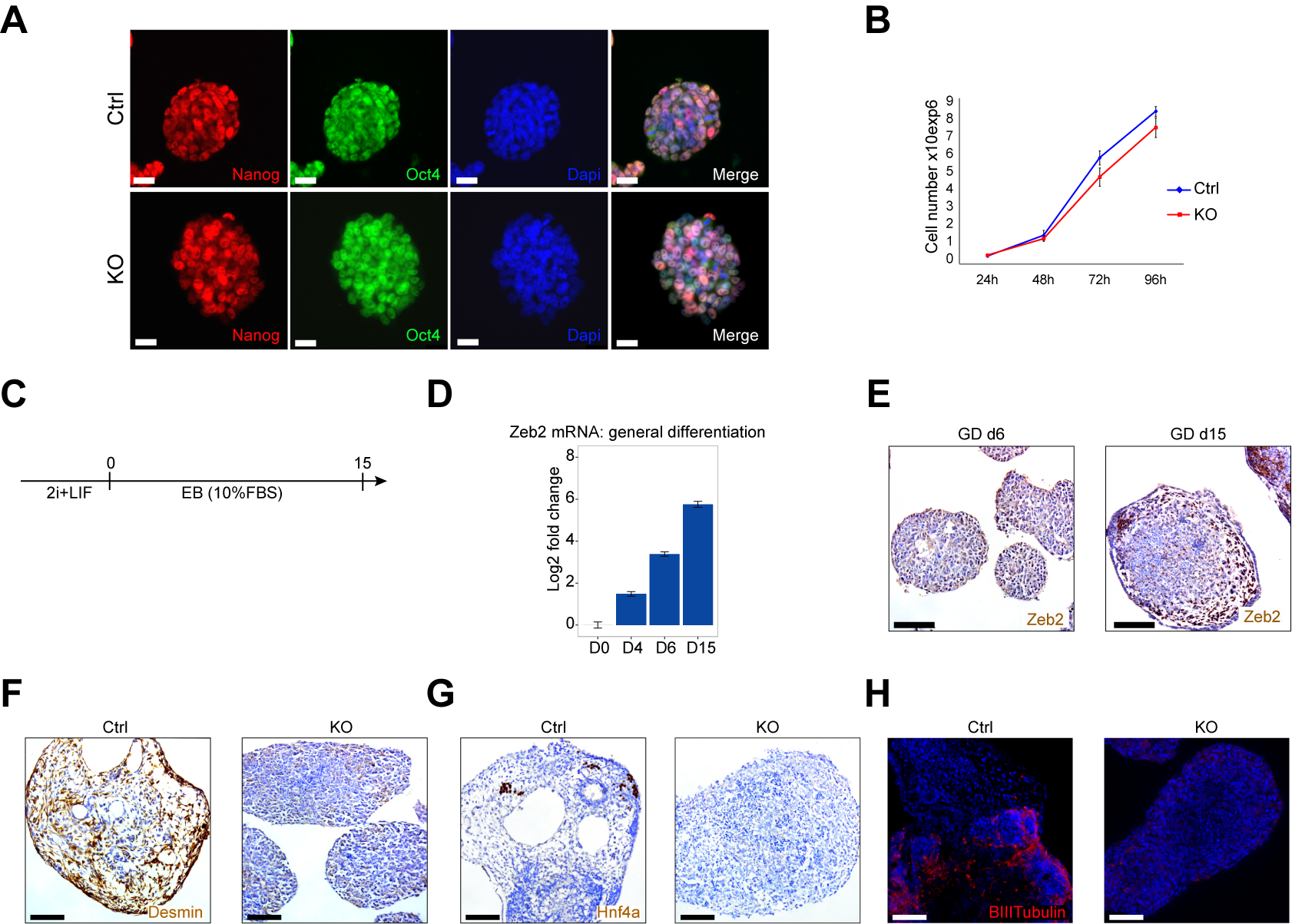

Supplement: Supplementary file 1 — Supporting Information Figure 1 [file STEM-35-611-s001.tif]

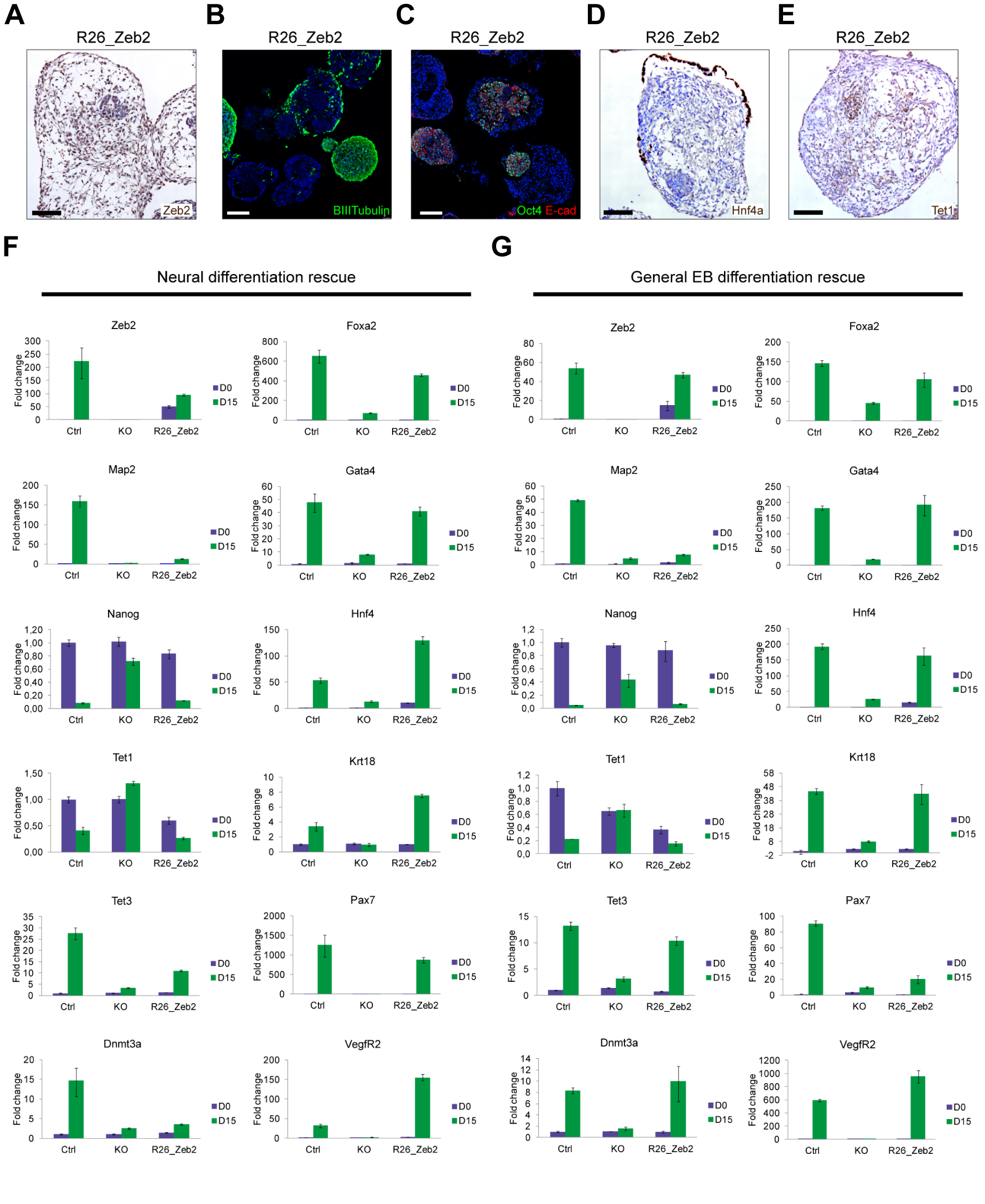

Supplement: Supplementary file 2 — Supporting Information Figure 2 [file STEM-35-611-s002.tif]

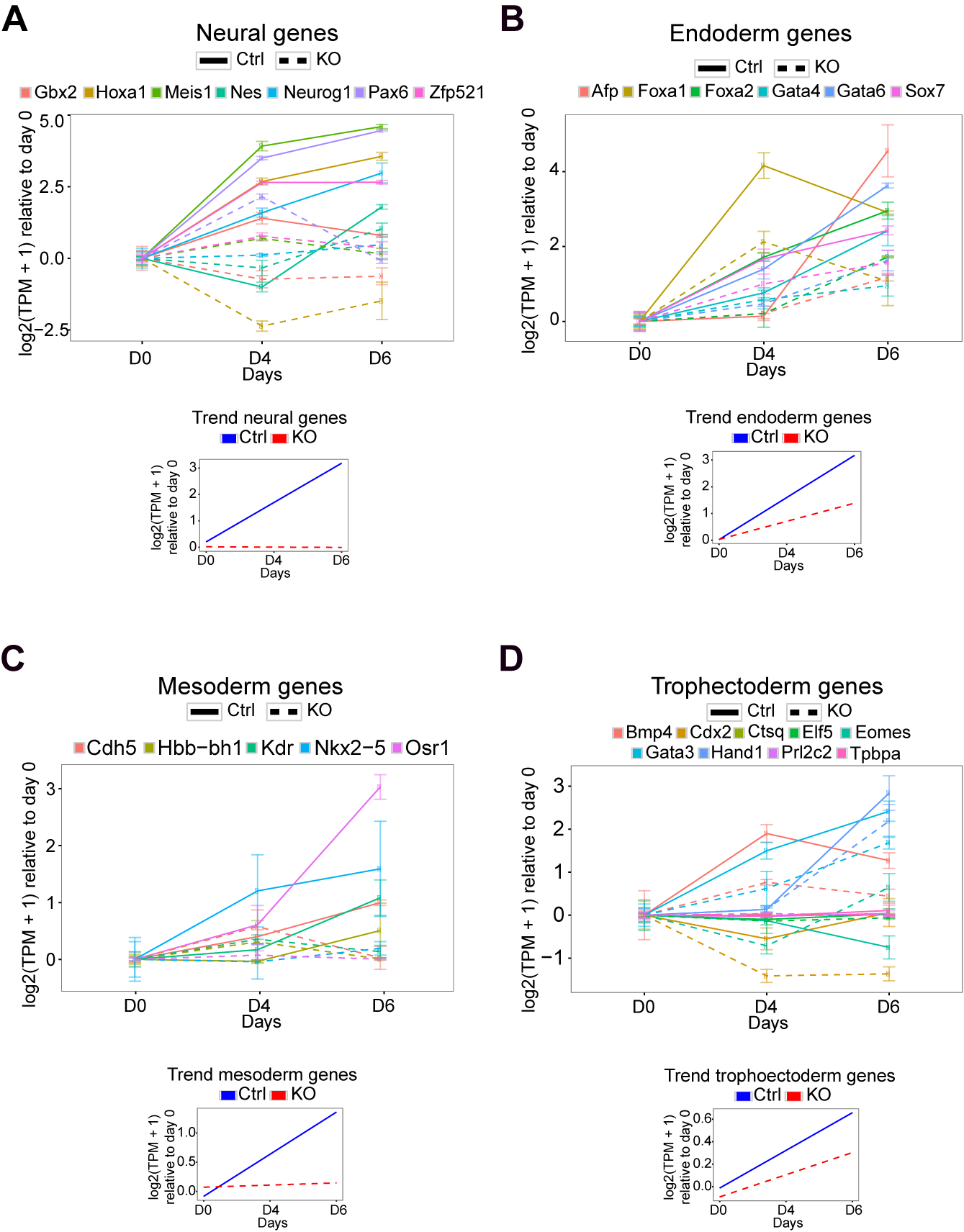

Supplement: Supplementary file 3 — Supporting Information Figure 3 [file STEM-35-611-s003.tif]

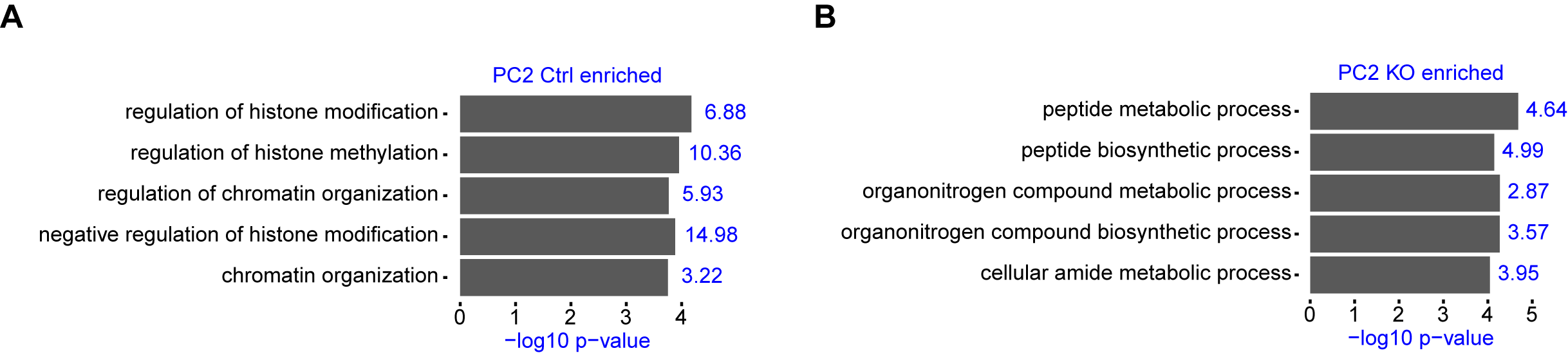

Supplement: Supplementary file 4 — Supporting Information Figure 4 [file STEM-35-611-s004.tif]

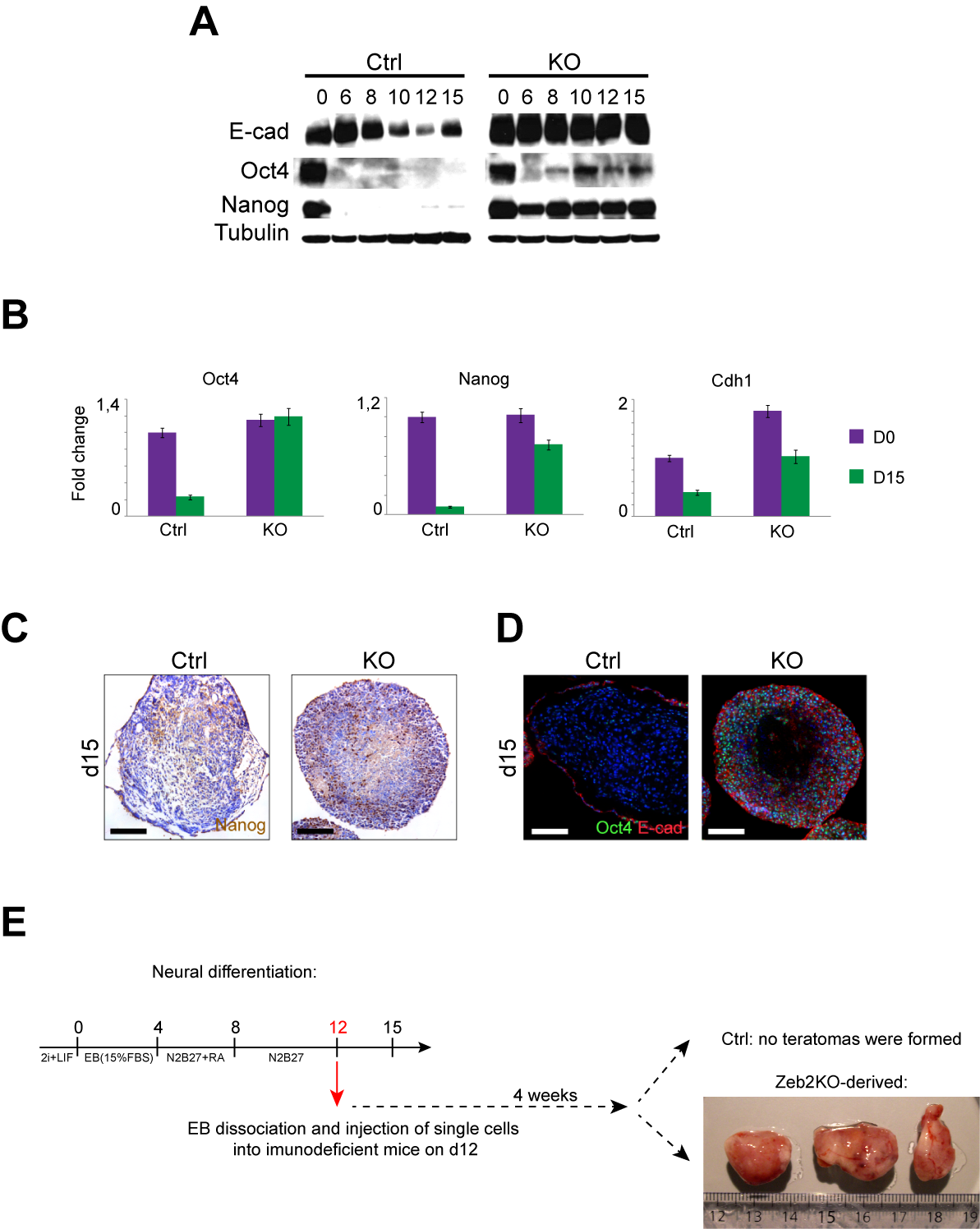

Supplement: Supplementary file 5 — Supporting Information Figure 5 [file STEM-35-611-s005.tif]

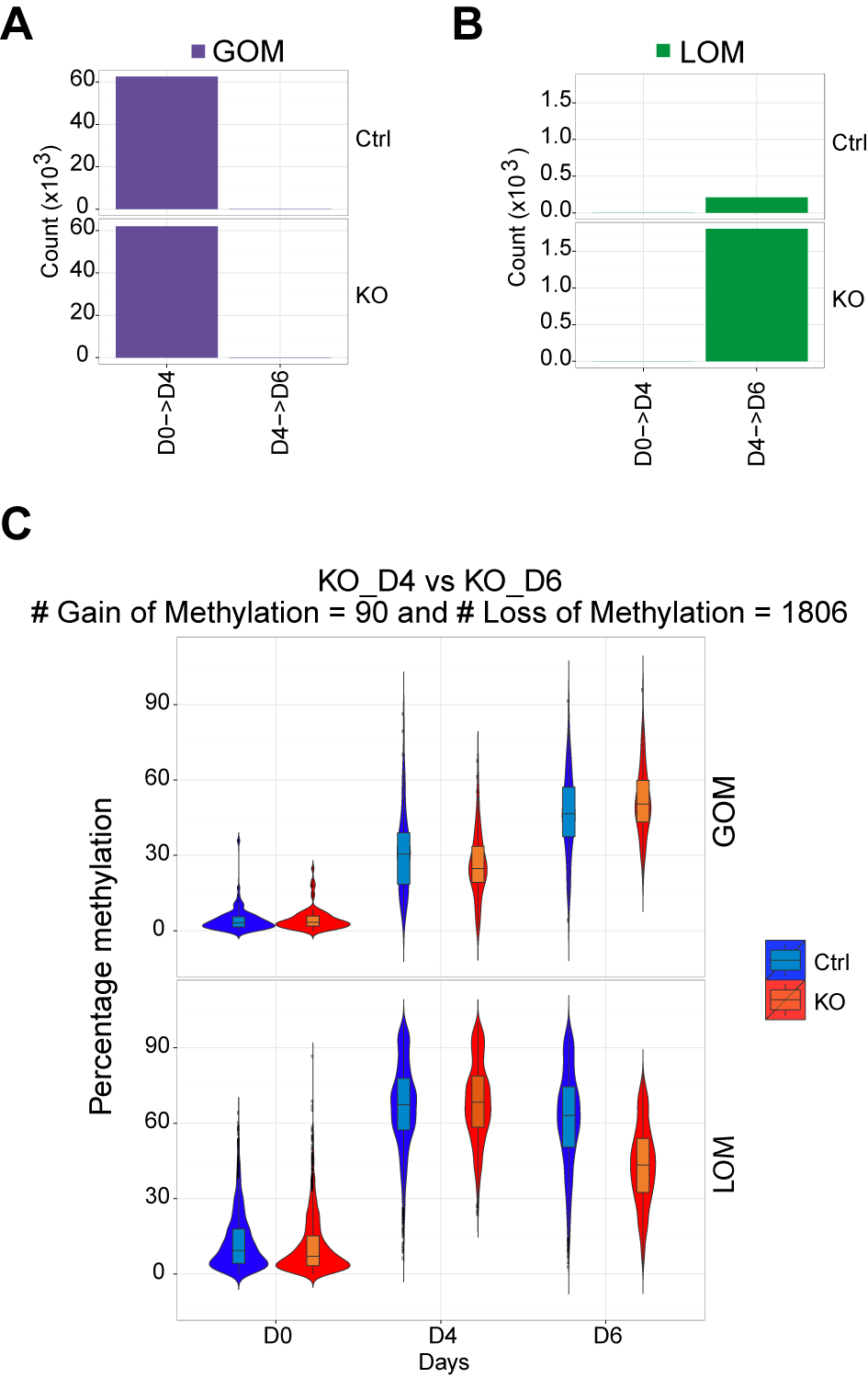

Supplement: Supplementary file 6 — Supporting Information Figure 6 [file STEM-35-611-s006.tif]

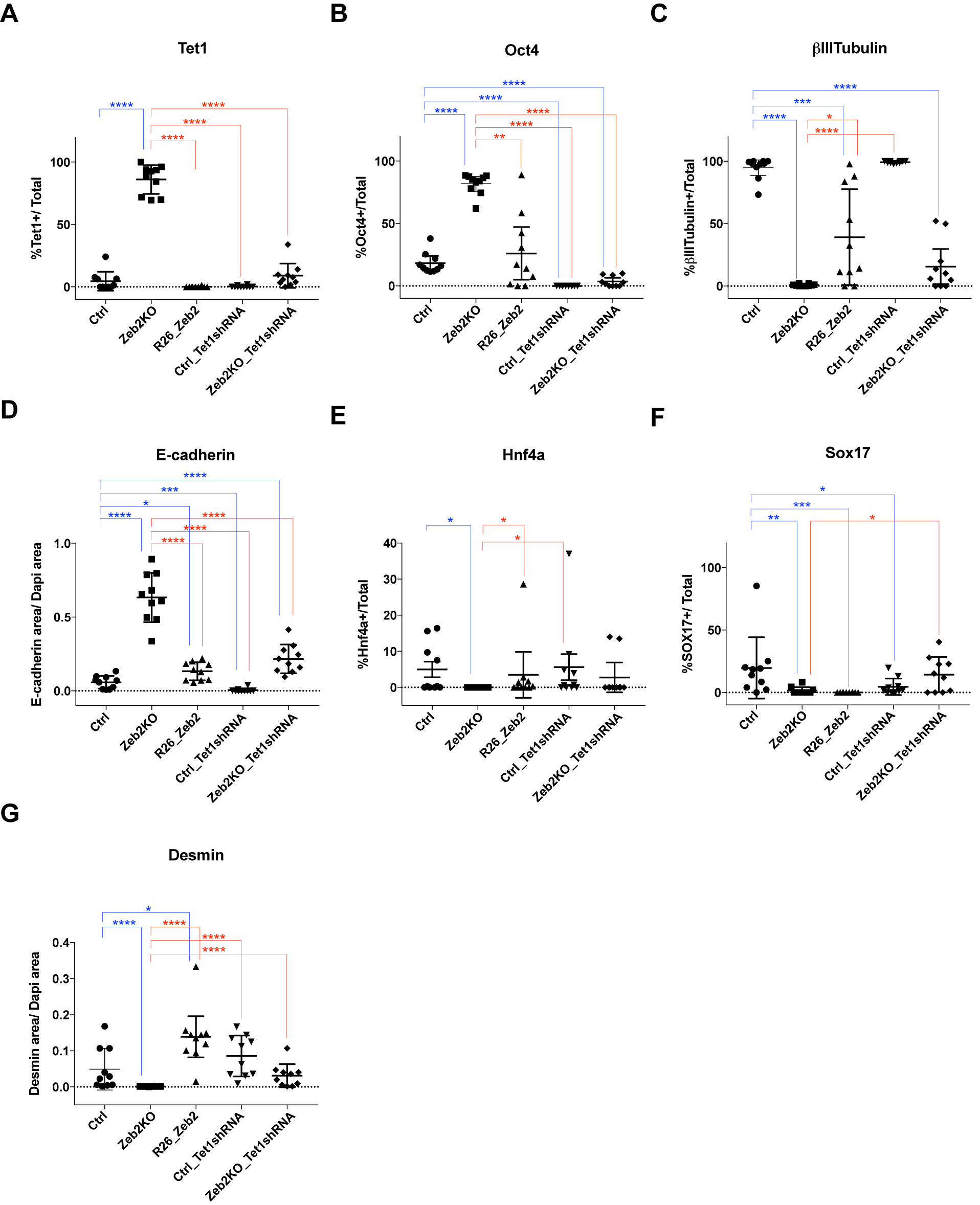

Supplement: Supplementary file 7 — Supporting Information Figure 7 [file STEM-35-611-s007.tif]
