# Supplementary material for: Zeb2 Regulates Cell Fate at the Exit from Epiblast State in Mouse Embryonic Stem Cells
Source: Stem Cells. 2016 Nov 8;35(3):611–25. doi: 10.1002/stem.2521 (PMC5396376; doi:10.1002/stem.2521)
Supplement: Supplementary file 8 — Supporting Information Tables 1‐2 [file STEM-35-611-s008.docx]

**Supplemental Tables I-II**

| Supplemental Table I: qPCR primers: | | |
| --- | --- | --- |
| Gene | Orientation | Sequence |
|  |  |  |
| Afp | Fwd | CTTCCCTCATCCTCCTGCTAC |
| Afp | Rev | ACAAACTGGGTAAAGGTGATGG |
| Cdx2 | Fwd | CAAGGACGTGAGCATGTATCC |
| Cdx2 | Rev | GTAACCACCGTAGTCCGGGTA |
| Cer1 | Fwd | CAACCACGAGGAGGCAGAAG |
| Cer1 | Rev | GATCGCTTTCCACATCCCTT |
| Chrd | Fwd | CTGCGCTCAAGTTTACGCTTC |
| Chrd | Rev | AGGGTGTTCAAACAGGATGTTG |
| Cxcr4 | Fwd | GACTGGCATAGTCGGCAATG |
| Cxcr4 | Rev | AGAAGGGGAGTGTGATGACAAA |
| Dnmt3a | Fwd | GAGGGAACTGAGACCCCAC |
| Dnmt3a | Rev | CTGGAAGGTGAGTCTTGGCA |
| Dnmt3b | Fwd | AGCGGGTATGAGGAGTGCAT |
| Dnmt3b | Rev | GGGAGCATCCTTCGTGTCTG |
| Eomes | Fwd | CCTGGTGGTGTTTTGTTGTG |
| Eomes | Rev | TTTAATAGCACCGGGCACTC |
| Esrrb | Fwd | TTAACGCCATCCCCAAGCGCC |
| Esrrb | Rev | CAAGGCGCACACCTTCCTTCAGC |
| Fillagrin | Fwd | ATGTCCGCTCTCCTGGAAAG |
| Fillagrin | Rev | TGGATTCTTCAAGACTGCCTGTA |
| Foxa2 | Fwd | TCCGACTGGAGCAGCTACTAC |
| Foxa2 | Rev | GCGCCCACATAGGATGACA |
| Gata4 | Fwd | CACCCCAATCTCGATATGTTTGA |
| Gata4 | Rev | GCACAGGTAGTGTCCCGTC |
| Gata6 | Fwd | TTGCTCCGGTAACAGCAGTG |
| Gata6 | Rev | GTGGTCGCTTGTGTAGAAGGA |
| Gfap | Fwd | CCAGATCCGAGAAACCAGCCTGGA |
| Gfap | Rev | TGAGGTGGCCTTCTGACACGGA |
| Gsc | Fwd | CCCCGGTTCTGTACTGGTG |
| Gsc | Rev | TCTGGGTACTTCGTCTCCTGG |
| Hnf4 | Fwd | CACGCGGAGGTCAAGCTAC |
| Hnf4 | Rev | CCCAGAGATGGGAGAGGTGAT |
| Krt1 | Fwd | TGGGAGATTTTCAGGAGGAGG |
| Krt1 | Rev | GCCACACTCTTGGAGATGCTC |
| Krt10 | Fwd | CGAAGAGCTGGCCTACCTAAA |
| Krt10 | Rev | GGGCAGCGTTCATTTCCAC |
| Krt14 | Fwd | GAGGAGACCAAAGGCCGTTAC |
| Krt14 | Rev | GAGGAGAATTGAGAGGATGAGGA |
| Krt18 | Fwd | GTCAGAGACTGGGGCCACTA |
| Krt18 | Rev | CTCTAAAGTCATCGGCGGCAA |
| Lefty1 | Fwd | CCAACCGCACTGCCCTTAT |
| Lefty1 | Rev | CGCGAAACGAACCAACTTGT |
| Lefty2 | Fwd | CAGCCAGAATTTTCGAGAGGT |
| Lefty2 | Rev | CAGTGCGATTGGAGCCATC |
| Map2 | Fwd | GCCAGCCTCAGAACAAACAG |
| Map2 | Rev | AAGGTCTTGGGAGGGAAGAAC |
| Mixl1 | Fwd | ACGCAGTGCTTTCCAAACC |
| Mixl1 | Rev | CCCGCAAGTGGATGTCTGG |
| Nanog | Fwd | TCTTCCTGGTCCCCACAGTTT |
| Nanog | Rev | GCAAGAATAGTTCTCGGGATGAA |
| Nodal | Fwd | TTCAAGCCTGTTGGGCTCTAC |
| Nodal | Rev | TCCGGTCACGTCCACATCTT |
| Oct4 | Fwd | AGAGGATCACCTTGGGGTACA |
| Oct4 | Rev | CGAAGCGACAGATGGTGGTC |
| Pax7 | Fwd | TCTCCAAGATTCTGTGCCGAT |
| Pax7 | Rev | CGGGGTTCTCTCTCTTATACTCC |
| Pdgfra | Fwd | TCCATGCTAGACTCAGAAGTCA |
| Pdgfra | Rev | TCCCGGTGGACACAATTTTTC |
| Pdgfrb | Fwd | TTCCAGGAGTGATACCAGCTT |
| Pdgfrb | Rev | AGGGGGCGTGATGACTAGG |
| Prdm14 | Fwd | CTCTTGATGCTTTTCGGATGACT |
| Prdm14 | Rev | GTGACAATTTGTACCAGGGCA |
| Sox17 | Fwd | GATGCGGGATACGCCAGTG |
| Sox17 | Rev | CCACCACCTCGCCTTTCAC |
| T | Fwd | CTCGGATTCACATCGTGAGAG |
| T | Rev | AAGGCTTTAGCAAATGGGTTGTA |
| Tet1 | Fwd | ACACAGTGGTGCTAATGCAG |
| Tet1 | Rev | AGCATGAACGGGAGAATCGG |
| Tet2 | Fwd | AGAGAAGACAATCGAGAAGTCGG |
| Tet2 | Rev | CCTTCCGTACTCCCAAACTCAT |
| Tet3 | Fwd | TCTCTGAAGGGTGGATTGTCC |
| Tet3 | Rev | CCCAGCACCGAGTAGCTTTC |
| VegfR2 | Fwd | TTTGGCAAATACAACCCTTCAGA |
| VegfR2 | Rev | GCAGAAGATACTGTCACCACC |
| Zeb1 | Fwd | ACCGCCGTCATTTATCCTGAG |
| Zeb1 | Rev | CATCTGGTGTTCCGTTTTCATCA |
| Zeb2 | Fwd | ACCTTACGAATGCCCAAACTGCA |
| Zeb2 | Rev | ACAGAATTAGGGGAAGAACCCGTCT |
| Zfp42 | Fwd | CCCTCGACAGACTGACCCTAA |
| Zfp42 | Rev | TCGGGGCTAATCTCACTTTCAT |
| Zfp521 | Fwd | GAGCGAAGAGGAGTTTTTGG |
| Zfp521 | Rev | AGTTCCAAGGTGGAGGTCAC |
|  |  |  |
| Supplemental Table II: qPCR ChIP primers: | | |
| Gene | Orientation | Sequence |
| Cdh1 Ctrl region | Fwd | TGAGGTCCTAGGTTCCATCTC |
| Cdh1 Ctrl region | Rev | GAAGGCAGGAACTGAACACA |
| Cdh R1 | Fwd | GCTAGGCTAGGATTCGAACGAC |
| Cdh R1 | Rev | TGCAGGGCCCTCAACTT |
| Nanog R1 | Fwd | CAGCCGTGGTTAAAAGATGAATAAAGTG |
| Nanog R1 | Rev | CAGCCGTGGTTAAAAGATGAATAAAGTG |
| Nanog Ctrl region | Fwd | GGTGATACGTTGGCCTTCTAGTCTGAA |
| Nanog Ctrl region | Rev | GGGCAAATTGCAAACTAACTGTATAACCTC |
