# Supplementary material for: Zeb2 Regulates Cell Fate at the Exit from Epiblast State in Mouse Embryonic Stem Cells
Source: Stem Cells. 2016 Nov 8;35(3):611–25. doi: 10.1002/stem.2521 (PMC5396376; doi:10.1002/stem.2521)
Supplement: Supplementary file 9 — Supporting Information Tables 2‐3 [file STEM-35-611-s009.docx]

**Supplementary Tables III-VII**

These tables can be uploaded as .xlxs files on the Stem cells webpage, but the conversion (of most of those) in pdf and incorporation in the manuscript result in a too large (>2000 pages !) file.

Hence, we left these Tables our from the manuscript for the time being and will in a next phase contact the journal to see how to proceed.

In particular, these are

**Suppl. Table III – PC1_PC2_genes**

**Suppl. Table IV – RNAseq – Time series**

**Suppl. Table V – RNAseq – Pairwise**

**Suppl. Table VI – RRBS – Pairwise**

**Suppl. Table VII – RRBS – Temporal**
