# Supplementary material for: Zeb2 Regulates Cell Fate at the Exit from Epiblast State in Mouse Embryonic Stem Cells
Source: Stem Cells. 2016 Nov 8;35(3):611–25. doi: 10.1002/stem.2521 (PMC5396376; doi:10.1002/stem.2521)
Supplement: Supplementary file 15 — Supporting Information Figure Caption [file STEM-35-611-s015.docx]

**Supplemental Figures**

**Supplemental Figure 1, related to Figure 1. Genetic inactivation of *Zeb2* in mouse ESCs does neither impair ESC proliferation nor Oct4/Nanog production in pluripotency-supporting conditions.** A. Control (Ctrl) and *Zeb2* knockout (KO) ESCs, grown in 2i+LIF, were co-stained by indirect IF for Nanog (red) and Oct4 (green). Results are representative of 3 experiments performed. Bar: 25 µm. B. Cell proliferation of Ctrl (blue) and *Zeb2* KO (red) ESCs grown in 2i+LIF. SDs are of 3 biological replicates. C. Scheme of the general differentiation (GD) protocol; FBS= fetal bovine serum). D. Zeb2 mRNA levels increased in Ctrl ESCs submitted to GD, albeit to lower relative levels as in ND (see Fig. 1B). E. Ctrl EBs were stained for Zeb2 (brown) on d6 and d15 of GD. Scale bars: 50 µm. F-H. IHC and IF analysis to document differentiation towards mesoderm (Desmin, panels F), endoderm (Hnf4α; panels G) and neural cells (βIIITubulin; panels H). Scale bars: 50 µm. Results shown are from one experiment and representative for 3 experiments. In contrast to Ctrl cells, very few Desmin+ cells were detected in *Zeb2* KO EBs after d15 in GD, while Hnf4α levels appear reduced in *Zeb2* KO EBs. The neural commitment defects first observed in ND were re-confirmed in GD as βIIITubulin+ cells were absent in *Zeb2* KO EBs.

**Supplemental Figure 2, related to Figure 1. The differentiation defects are rescued in R26_Zeb2 ESC lines.** A-E. IHC and IF of R26_ZEB2 line after 15 days of general (GD) or neural (ND) differentiation A. Zeb2 (brown) in GD. B. βIIITubulin (green), ND. C. Oct4 (green) and Cdh1 (red), ND. D. Hnf4a (brown), GD. E. Tet1 (brown). Scale bar: 50 μm. F. RT-qPCR for marker genes representative for three-lineage differentiation, pluripotency and DNA-methylation, respectively, in Ctrl, *Zeb2* KO and R26_Zeb2 lines on d0 (blue) and 15 (red) of ND. Results are representative of 3 experiments performed. SD of 2 technical replicates is shown. G. RT-qPCR for marker genes (see above) in Ctrl, *Zeb2* KO and R26_Zeb2 lines on d0 (violet) and 15 (green) of GD. Results are representative of 3 experiments performed. SD of 2 technical replicates is shown.

**Supplemental Figure 3**. Dynamic expression levels in Ctrl and *Zeb2* KO cells for selected neural (A) endoderm (B), mesoderm (C) [and trophectoderm (D), see text] genes at three stages in ND (d0, d4, d6, respectively). Average trend for selected genes in Ctrl and KO cells is modeled by simple linear regression line.

**Supplemental Fig. 4, related to Figure 2.** **Analysis of temporal RNA-seq**. A. GO analysis for top-100 genes in the PC2 in the Ctrl cells. B. GO analysis for top-100 genes in the PC2 in the *Zeb2* KO cells.

**Supplemental Figure 5, related to Figure 4**. **Pluripotency gene expression is not silenced during differentiation in *Zeb2* KO cells**. A. Western blot analysis for Cdh1, Oct4 and Nanog in Ctrl and *Zeb2* KO (KO) EBs during ND. B. RT-qPCR analysis of in Ctrl and KO ESCs (D0) and EBs on d15 of ND for Oct4, Nanog and Cdh1. Results are representative of 3 experiments performed. SD of 2 technical replicates is shown C. Ctrl and *Zeb2* KO EBs stained for Nanog (brown) on d15 of GD D. Ctrl and KO EBs co-stained for Oct4 (green) and Cdh1 (red) on d15 of GD. Panels C-D show results from one experiment that is representative for 3 experiments. Scale bar: 50 µm E. Teratoma formation assay with EBs subjected to ND for 12 days.

**Supplemental Figure 6, related to Figure 5. RRBS.** A-B. Bar plot showing gain (GOM, panel A) and loss-of-methylation (LOM; panel B) between consecutive time points in Ctrl and *Zeb2* KO ESCs. C. Violin plots to illustrate distribution and dynamic behavior of DMRs (both GOM and LOM) over time.

**Supplemental Figure 7, related to Figures 1 and 6. Tet1 knockdown in *Zeb2* knockout ESCs facilitates their definitive pluripotency exit and partially restores their neural (ND) and general differentiation (GD) defect.** Quantifications of staining of EBs (each EB indicated as individual data point): A. Tet1 on d15 of GD, B. Oct4 on d15 of ND, C. βIIITubulin on d15 of ND, D. Cdh1 on d15 of ND, E. Hnf4a on d15 of GD, F. Sox17 on d15 of GD, G. Desmin on d15 of GD. Statistical analysis: Non-parametric Mann-Whitney test. Error bars show mean with 95% confidence intervals. p-val: ****: <0.0001, ***:<0.001, 0.01<**<0.05:, *<0.05.
